# Comparison of Artificial Intelligence Tools With Human Coding for Sentiment, Topic, and Thematic Analysis Tasks of Public Health Datasets During the COVID-19 Pandemic in Australia: Case Study

**DOI:** 10.2196/80824

**Published:** 2026-04-07

**Authors:** Danielle Hutchinson, Lauren Lee, Haley Stone, Aye Moa, Holly Seale, C Raina MacIntyre

**Affiliations:** 1 Biosecurity Program, Kirby Institute, Faculty of Medicine and Health, UNSW Sydney, Wallace Wurth Building, Sydney, 2052, Australia; 2 School of Population Health, Faculty of Medicine and Health, UNSW Sydney, Sydney, Australia

**Keywords:** public opinion, sentiment analysis, social media, artificial intelligence, AI, public health informatics, equity, COVID-19

## Abstract

**Background:**

Public opinion, which may be influenced by personal experiences, news, and social media, can impact compliance with public health measures (PHMs) during health emergencies. Artificial intelligence (AI) tools offer opportunities to analyze public opinion in real time during health emergencies. However, their performance in accurately identifying sentiment and themes in health-related online content remains unclear.

**Objective:**

This study aimed to evaluate the performance of natural language processing–based and large language model (LLM)–based AI tools when compared to human coding for sentiment analysis, topic modeling, and thematic analysis of public health datasets. Tools were selected to reflect those available to public health analysts and decision-makers.

**Methods:**

Data were collected via Google Alerts (GA) and social media posts from X (formerly known as Twitter) relevant to COVID-19 mitigation PHMs from December 2022 to February 2023. Following relevance screening, the sentiment of the complete datasets was analyzed by a human rater, with descriptive statistics used to summarize the overall sentiment profile. Subsets of 400 GA articles and 400 tweets were manually coded for sentiment by 2 human raters. Results were compared with outputs from 5 AI tools, including VADER (Valence Aware Dictionary and Sentiment Reasoner), SentimentGI, SentimentQDAP, Microsoft Azure, and OpenAI’s ChatGPT-4. Topic modeling of the GA and X datasets was conducted using latent Dirichlet allocation in R and zero-shot prompting in ChatGPT-4 and compared with manual topic summaries. Thematic analysis of positive and negative sentiment datasets was conducted by a human rater and ChatGPT-4, with outputs evaluated for proficiency and reasonableness. The sentiment of the entire datasets was analyzed by a human rater, and descriptive statistics were calculated.

**Results:**

Of 2227 GA results and 3484 tweets, 58% (n=1238) and 71% (n=2473), respectively, were relevant to PHMs. Human-coded sentiment analysis showed mostly neutral reporting in the news media, while social media expressed more polarized views. Across both datasets, AI tools demonstrated poor concordance with human-coded sentiment (Cohen κ <0.5 for all tools and sentiment categories). Topic modeling with ChatGPT-4 aligned more closely with human-rated topics than latent Dirichlet allocation, and of the 20 LLM-generated thematic outputs, 13 were rated proficient, and 7 were rated partially proficient. LLM outputs provided coherent, high-level summaries but lacked contextual insight. Human and LLM thematic analyses both identified themes of vaccine effectiveness, debate regarding PHMs, and public trust.

**Conclusions:**

Accessible AI tools demonstrate limited reliability for sentiment classification of health-related online text but show promise for rapid thematic exploration when combined with human oversight. These tools could complement traditional qualitative research in the context of health emergencies; however, they require human review to enhance the accuracy of interpretation. Further research is needed for non-English datasets.

## Introduction

In the context of health, public opinions can change over time, vary across the population, and are often influenced by factors such as personal experiences and media exposure [1]. Public opinion can impact the course of an epidemic through impacting levels of compliance with public health measures (PHMs) such as vaccines, mask-wearing, and social distancing [2]. For example, Yu et al [3] used agent-based modeling to describe the relationship between the spread of COVID-19 and opinion dynamics in 15 different countries and found that public opinion on preventive interventions impacted the cumulative number of cases, particularly in the early stages of an epidemic. The World Health Organization has identified disinformation, the intentional spread of misleading information, as a threat to public health [4]. This may occur by changing the opinions and, therefore, the behaviors of populations through the creation of uncertainty about PHMs [4,5]. Therefore, the collection of public opinion data is important to measure public acceptance of PHMs and to monitor changes over time, thereby serving as a tool to combat the impact of disinformation and promote compliance with public health advice. There has been a proliferation of published research using artificial intelligence (AI) tools to analyze these data [6]. However, there is limited understanding of the accuracy of these readily available AI tools in conducting sentiment, topic, or thematic analysis on datasets of public opinion in real-world scenarios.

Public opinion data provide an important feedback mechanism during health emergencies such as the COVID-19 pandemic [7]. Qualitative research methods, such as analysis of data collected from focus groups and individual interviews, are recommended to better understand community opinions about disease perception and preventive behaviors to inform response efforts during health emergencies [8-10]. However, barriers to rapid qualitative research in the context of an infectious disease outbreak may include difficulties in conducting focus groups and interviews due to exposure risks, and participants may be reluctant to participate in the study due to the impact of the disease or the public health response [8,10]. In emergency situations, it is important to share findings in almost real time; therefore, the time taken to conduct qualitative research is a barrier, particularly during health emergencies [8,10]. Rapid data analysis techniques may deliver some time savings; however, data collection, interpretation, and write-up of results remain time intensive [11], with the World Health Organization European guidelines suggesting that the entire process takes 4 to 6 weeks, and Dong et al [12] classified rapid qualitative methods as those taking less than 6 months from conception to reporting of results [9,12]. Comparatively, data collection and analysis using AI can take as little as a few minutes [13]. Other barriers may include the capacity to form a research team of available staff with the necessary expertise to undertake qualitative analysis of community opinion to inform health policy [8].

Health emergencies such as the COVID-19 pandemic have led to the consideration of new methodologies for the collection and use of evidence to inform policy decision-making [14]. Previous studies have proposed the use of AI tools, such as natural language processing (NLP) and large language models (LLMs), to reduce the workload and provide real-time insights to help inform public health decision-making [15-19]. AI tools can be used to complement traditional qualitative data analysis methods in public health through sentiment analysis, topic modeling, and thematic analysis and have been shown to be less time-consuming and resource-intensive for these tasks [13,20-23]. Access to platforms to perform social media analysis, as well as improved training and capacity to conduct this type of research, was identified as key areas for improvement in a global survey of public health professionals [24]. Our study has specifically chosen tools that are readily available and do not require in-depth training for a public health professional to use.

Despite the proliferation of published research on public opinion data, collected via surveys and social media, toward PHMs during the COVID-19 pandemic, it is unclear whether these data are used as part of evidence-based policy decision-making [25].

This study adopted an applied informatics perspective, focusing on tools and data pipelines readily available to Australian public health analysts. The study aimed to evaluate the feasibility and performance of commonly available AI text analysis tools in analyzing public health datasets from social media (X) and news media (Google Alerts [GA]). Specifically, the study examined how traditional NLP methods and LLM tools perform when analyzing online public opinion data about PHMs during the COVID-19 pandemic in Australia.

The study addressed two research questions:

1.  How accurately and consistently do AI-based tools classify sentiment and extract relevant topic and thematic content from public health–related online text?
2.  What role does human oversight play in ensuring the validity and reliability of AI-driven sentiment and thematic analysis in public health informatics workflows?

First, we hypothesized that AI tools would demonstrate generally limited accuracy for broad sentiment classification and that LLMs would perform better than NLP-based text analysis tools in identifying and summarizing thematic content. Second, we hypothesized that human review and interpretive oversight will remain necessary to ensure data quality and contextual understanding of online public health datasets, underscoring the continuing importance of human oversight in public health informatics workflows.

## Methods

### Data Sources and Sampling

This study used open-source online data to evaluate the feasibility of automated methods for analyzing public opinion about PHMs during the COVID-19 pandemic in Australia. Two platforms were selected: GA and X (formerly known as Twitter), representing traditional and social media sources, respectively, and the most commonly used sources in infodemiology research [26].

GA is a change detection and notification system that automatically monitors multiple websites for mentions of a textual string and allows the user to select the frequency of monitoring, the source, language, and region [27,28]. GA was configured to retrieve Australian news articles that included key COVID-19–related terms (Table 1) once daily [28]. Each alert provided the publication date, publisher, article title, and 2-line summary, which were collected and stored in a Microsoft Excel spreadsheet. Data collection occurred from December 19, 2022, to February 19, 2023, during the fourth Omicron COVID-19 wave in Australia [29].

**Table 1.** Search terms.

Public health measure	Search terms
Vaccination	Vaccine, booster, vax, jab, Pfizer, Moderna, Novavax, (kids OR children) AND (Vaccine)
Mask-wearing	Mask, n95 OR respirator, school AND masks, (kids or children) AND (mask or unmask), public transport AND mask
Lockdown	Lockdown
Isolation requirements	Isolation, iso
Mandates	Mandate
Ventilation	Ventilation

The social media platform X is one of the most widely used platforms for health research and was freely available with an academic license at the time of data collection [30,31].

A Python app was used to access the X academic application programming interface (API) and to search for tweets geolocated in Australia using identical keyword combinations and date ranges. Specifically, we used a custom script to handle query construction, data collection, and filtering by keywords, language, and location. Separate searches were conducted for each keyword. All English-language tweets posted in Australia on these days were retrieved and included in the relevance assessment. Tweets with geolocation outside Australia and non–English-language tweets were excluded. Tweet ID and date of publication, tweet content, region, and location were collected and stored in a Microsoft Excel spreadsheet. User information was not collected to maintain anonymity.

A random sample of 800 items (400 GA articles and 400 tweets) was drawn using the RAND() function in Microsoft Excel (version 2306; Microsoft 365) to produce a manageable dataset for manual validation and automated analysis [32]. This dataset size allowed comparison across multiple sentiment and thematic analysis tools while maintaining a feasible human coding time for the manually coded reference set.

### Data Cleaning and Preprocessing

Text data from both platforms were preprocessed to standardize the format and remove noise before analysis. Duplicates were removed from the GA dataset by checking for duplicate URLs, thereby retaining articles with similar content published across different days or in different publications (eg, syndicated articles). Within the X dataset, duplicate tweets were removed, while quote tweets and retweets were preserved for analysis. This allowed for analysis of the volume of public interest across news media and social media regarding the topics of interest. Data preprocessing included the removal of Twitter handles, URLs, stop words, and punctuation. Individual GA articles and tweets that discussed more than one PHM (eg, both mask-wearing and vaccination) were disaggregated and analyzed separately for each relevant PHM category.

All collected GA results and tweets were assessed for relevance to PHMs as per the inclusion and exclusion criteria (Textbox 1) by 1 reviewer (DH). A subset of 100 tweets and 100 news articles was assessed by a second reviewer (HS), and interrater reliability was assessed using the Cohen κ coefficient [33].

Textbox 1. **Inclusion and exclusion criteria for relevance of articles to public health measures.**

Inclusion criteria

- Subjective (opinions, editorial, and commentary) or objective (information and policy announcements) discourse related to pharmacological (vaccines or other) and nonpharmacological (masks, ventilation, physical distancing, stay-at-home orders or lockdowns, and school closures) public health measures for communicable diseases (any).
- General information about vaccines

Exclusion criteria

- Scientific or medical journal papers or reports of the same
- Articles about case numbers or modeling
- Articles not relating to communicable disease
- Articles not relating to public health measures: pharmacological (vaccines or other) and nonpharmacological (masks, ventilation, physical distancing, stay-at-home orders or lockdowns, and school closures)

### Human-Coded Sentiment Analysis

Human-coded sentiment analysis involves individuals manually reviewing textual data and assigning a sentiment label to each text based on its content [34]. A data subset comprising 400 GA and 400 tweets was randomly selected using the RAND() function in Microsoft Excel and reviewed by 2 reviewers (DH and LL). As only the first 2 sentences of the news article were included in the GA dataset, the search term was also provided to assist the reviewer to determine sentiment. Sentiment toward the PHM was assessed and assigned a positive, negative, or neutral label based on the interpretation of the text. If the sentiment toward the PHM was positive, but the sentiment of the whole tweet was negative, it was assigned a positive value.

The reviewers used a rule-based process with the development of annotation guidelines (Textbox 2) and met after the first 50 items to review the guidelines and discuss any clarifications needed. Reviewers met again at the completion of the analysis to address any ambiguous cases. Interrater reliability was calculated using the Cohen κ coefficient [33]. Disagreements between human raters were resolved through discussion and consensus to ensure consistency in the final labeled dataset used for AI comparison. One reviewer (DH) completed the sentiment annotation of the remaining relevant GA and X datasets. GA results and tweets that did not contain sufficient information to assign sentiment were removed from the datasets. GA results and tweets that discussed more than one PHM were analyzed separately for each relevant PHM category, allowing for assessment of sentiment associated with specific PHMs within the same source text. GA results and tweets labeled positive, negative, and neutral were aggregated for analysis. Search terms relating to vaccination and mask-wearing (Table 1) were combined for further analysis; for example, the results for “vax,” “jab,” and “vaccine” were collapsed under the umbrella term “vaccine.” The proportion of sentiment, expressed as a percentage of the total number of GA results and tweets, was calculated with 95% CIs.

Textbox 2. **Annotation guidelines for human-coded sentiment analysis of articles and tweets.**

Positive sentiment

- Positive words referencing the public health measure
- Criticism of opinions against the public health measure (eg, criticism of antivaxxers was assigned a positive sentiment toward vaccines)

Neutral sentiment

- Neutral reporting of events and announcements
- Mention of public health measures without expressed sentiment or opinion

Negative Sentiment

- Negative words referencing the public health measure
- Criticism of opinions supporting the public health measure

### AI Tools Evaluated for Sentiment Analysis Task

A total of 5 AI-based text analysis tools were evaluated to compare the performance of traditional NLP techniques and LLM-based methods, which were validated against human-coded reference data. Tools were selected based on their accessibility for public sector analysts and compatibility with existing Australian government data science infrastructure [35-38]. R (version 4.3.1; R Foundation for Statistical Computing) within RStudio (version 2023.06.1; Posit Software), sentiment analysis tools (Valence Aware Dictionary and Sentiment Reasoner [VADER], SentimentGI, and SentimentQDAP), Microsoft Azure Machine Learning, and ChatGPT-4 represent platforms that are free, low cost, or integrated within standard analytics environments used by public health agencies [39-43]. Each text entry was assigned a compound sentiment score ranging from −1 (most negative) to +1 (most positive), which was then categorized as positive, neutral, or negative using default thresholds (≥0.05 positive; ≤−0.05 negative).

### NLP Tools in R

R is used extensively in public health research and includes packages specifically designed for cleaning and visualizing large public health datasets [35].

VADER is a lexicon- and rule-based sentiment analysis tool optimized for social media text. It was implemented in R using the vader package [44].

The SentimentAnalysis package (SentimentGI and SentimentQDAP) in R extends lexicon-based sentiment scoring by incorporating valence shifters (eg, negators and amplifiers) that adjust for linguistic nuance, which makes it particularly suited to conversational or social media–style text [45].

### Microsoft Azure

Microsoft Azure Machine Learning Text Analytics (version 3.1; 2023) was used to assess sentiment through integration into the Microsoft Excel spreadsheet environment via the Power Query function, with results returned as sentiment probabilities for positive, neutral, and negative categories [46]. The Microsoft suite is widely used across Australian government agencies and was therefore included in this study [38]. API version and date of access were documented to ensure reproducibility.

### OpenAI ChatGPT (GPT-4)

ChatGPT was tested as an emerging LLM approach for qualitative text classification. Using OpenAI’s API (March 2024) [47], each text item was submitted with a structured zero-shot prompt (Table S1 in Multimedia Appendix 1). Responses were parsed programmatically for sentiment and theme. This approach reflects the real-world use of generative AI in rapid public health analysis while maintaining reproducibility through prompt documentation.

### Topic and Thematic Analysis

To demonstrate how these data could be used in the public health domain for decision-making and evaluation, topic summary and thematic analyses were performed. Topic summary analysis was performed on the entire dataset by identifying key phrases and trends manually by highlighting recurring words, phrases, or patterns that may represent shared topics within the datasets [48]. For comparison, topic modeling was conducted on the GA and X datasets using latent Dirichlet allocation (LDA), a probabilistic modeling technique used to identify topics occurring in a textual dataset [22]. This was done using the “topicmodels” package in R [49], and the code is included in Multimedia Appendix 2. ChatGPT-4 was used to replicate the manual topic selection using a zero-shot prompting technique outlined in Table S1 in Multimedia Appendix 1 [50]. Results were collated, and the top 5 topics from each method were compared.

For the thematic analysis, the positive and negative results in the GA and X datasets were extracted from the entire annotated dataset and put into 4 separate files. Neutral sentiment results were excluded. A qualitative thematic analysis explored the opinions expressed toward PHMs within the Australian news media and among X users. This was done using Braun and Clarke’s [51] 6-step process, coding each data line prior to identifying common themes across the dataset. Examples from the datasets were collated, with paraphrasing of tweets to maintain user anonymity. For comparison, ChatGPT-4 was used to replicate the manual thematic analysis [52] on the 4 datasets (GA positive, GA negative, X positive, and X negative results) using the zero-shot prompting technique outlined in Table S1 in Multimedia Appendix 1 [50]. The thematic analysis results were presented with subthemes and paraphrased representative sentences for each dataset.

### Statistical Analysis

Concordance of each AI tool with human sentiment analysis was calculated by comparing the rating of a subset of tweets and articles with the human-rated sentiment analysis. Interrater reliability was calculated using the Cohen κ coefficient [33]. The κ statistic was used to assess interrater agreement, and results were classified as 0 to 0.5 weak, 0.51 to 0.8 moderate, and 0.81 and above strong [33]. Statistical analysis was performed using R, where a 2-sided *P* value of <.05 was considered statistically significant [49].

### Validation and Comparison

All tool outputs were compared against the manually coded dataset. Descriptive and statistical analyses were performed on the results of the sentiment analysis process using Microsoft Excel [32]. For each sentiment (eg, “positive”), we identified all cases where the human rater assigned that sentiment and then calculated the percentage of those cases in which the AI tool assigned the same sentiment (Table S2 in Multimedia Appendix 1). This process was repeated for each sentiment category (positive, neutral, and negative) and each tool.

All statistical analyses were performed using R [53].

The accuracy of the generative AI results for topic modeling was calculated using the number of agreements in each dataset [52]. This was done using a cross-matching rubric (Table 2).

**Table 2.** Cross-matching rubric for alignment of human and artificial intelligence topic modeling and thematic analysis results.

Tasks and score	Label	Definition
Topic modeling		
2	Direct match	Strong alignment with any manually generated topic. Captures core meaning.
1	Partial match	Aligns with a related concept, but not the central idea. May be too generalized.
0	No match	Does not align with any manually generated topic.
Thematic analysis: proficiency		
—^a^	Proficient	The LLM-generated^b^ theme matched closely and accurately with high relevance to the human coding.
—	Partially proficient	The theme matched moderately well, with some relevance to the human coding.
—	Not proficient	The LLM theme matched poorly with little relevance to the human coding.
Thematic analysis: reasonableness		
2	Very reasonable	The theme had high relevance to the dataset and would likely be generated by human coding.
1	Reasonable	The theme had some relevance to the dataset and could be generated by human coding.
0	Not reasonable	The LLM-generated theme had little relevance to the dataset.

a Not applicable.

b LLM: large language model.

A comparative analysis of the human and LLM thematic analyses was conducted by 1 author (DH), who reviewed each output of the LLM and assessed whether the theme matched the human thematic analysis. A cross-matching rubric, similar to the one used for topic analysis agreement, was developed to classify the LLM-generated output as “proficient,” ”partially proficient,” or ”not proficient” in capturing themes from the data that matched the human coding (Table 2) [50,52]. For all LLM outputs, regardless of whether they matched the human output, a score for how “reasonable” it was to derive the theme from the dataset was given, using a scale of 0 to 2, as has been used in similar studies [52]. The scoring was completed by 1 author, a subject matter expert (DH; Table 2).

### Ethical Considerations

Nonidentifiable data from online news media and social media were collected during this study. We did not analyze individual accounts and have not published any identifiable information or individual quotes. The LLM used in this study, ChatGPT-4, was used in a strictly limited capacity for analyzing nonsensitive, deidentified text. The research team ensured that no identifiable data were shared with the AI platform, and all use complied with institutional research integrity and data privacy guidelines. All data were deidentified (including the removal of Twitter handles) prior to input, and chat history was disabled to prevent storage or reuse of content by the model provider. Chats were deleted when the session was complete. The study was approved by the UNSW Human Research Ethics Committee (approval number HC230028).

## Results

### Overview

The results of this study are presented as follows. First, a description of the dataset is given. Second, the results of the comparison of human-coded and machine-coded sentiment analysis on the data subset are outlined. Third, a summary of the sentiment analysis of the entire dataset is provided. Finally, human-generated and machine-generated topic modeling and thematic analysis results of the entire dataset are compared to demonstrate the feasibility of using AI tools to assist public health analysts in assessing public opinion during health emergencies.

### Description of the Dataset

Overall, 2761 GA articles and 3495 tweets were collected during the study period. Following removal of duplicates, 2227 GA articles and 3484 tweets were included in the relevance screening, with 57.6% (n=1283) of GA articles and 71% (n=2473) of tweets assessed as relevant. There were moderate (GA: 88% concordance, ĸ=0.76) and strong (X: 94% concordance, ĸ=0.86) levels of agreement for relevance between 2 analysts in the data subsets of 100 (88%) GA articles and 100 (100%) tweets.

**Figure 1.**
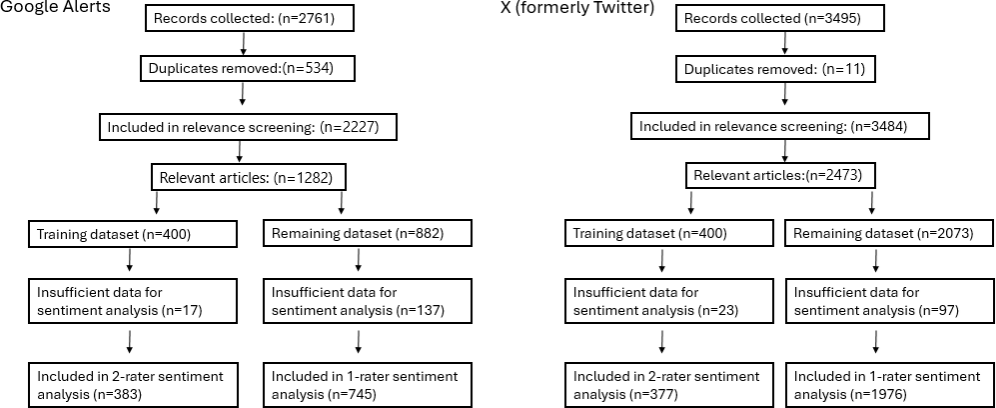
Flow diagram of included Google Alerts and X results.

During the sentiment analysis process, further GA articles (n=154) and tweets (n=120) were removed from the dataset, as there was not enough information to determine sentiment (Figure 1). There were moderate levels of agreement for sentiment scores between the 2 reviewers in both the GA dataset (κ=0.69; *P*<.001) and the X dataset (κ=0.75; *P*<.001; Table S2 in Multimedia Appendix 1).

### Sentiment Classification Performance of AI Tools

**Figure 2.**
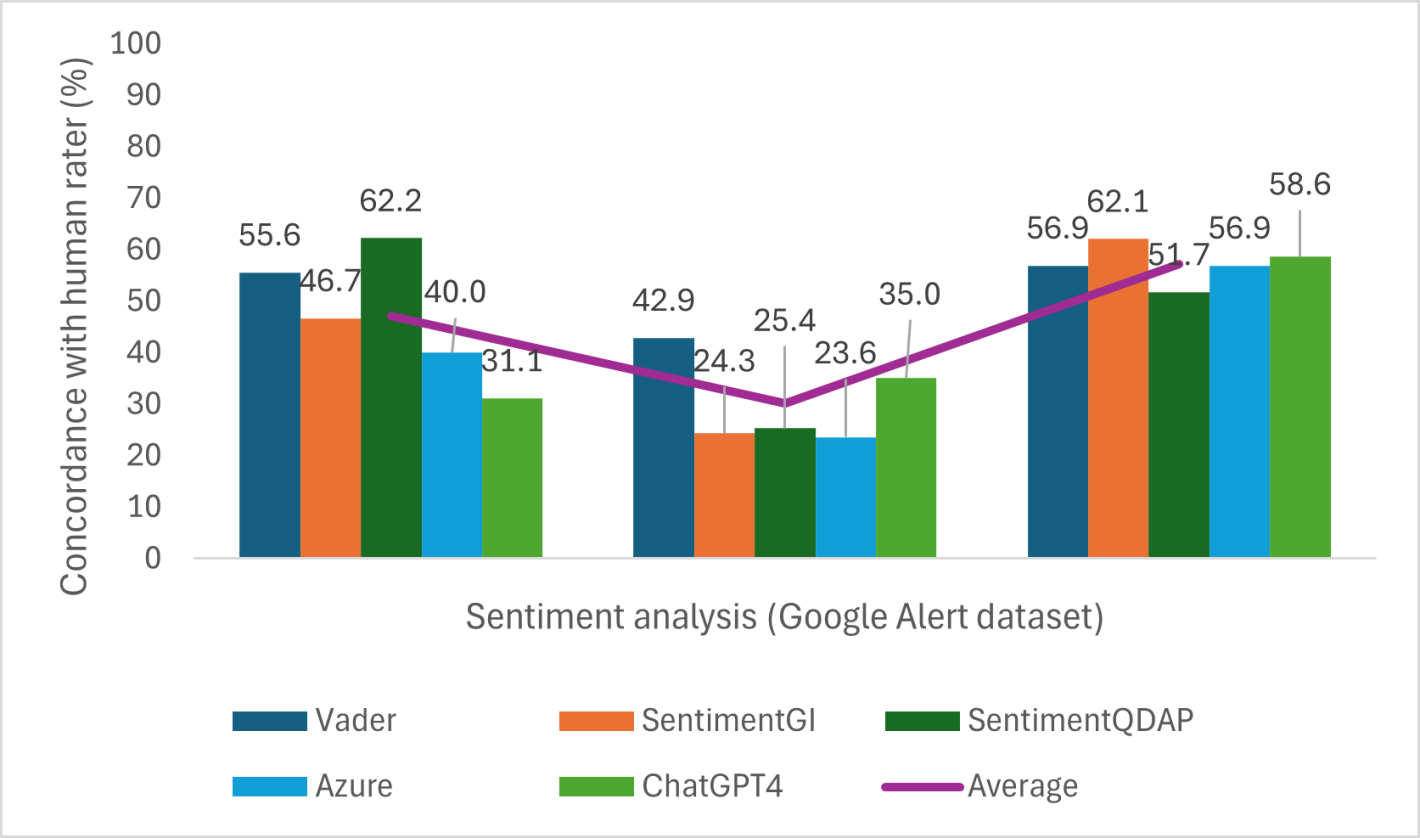
Comparison of human-rated sentiment score for a subset of Google Alerts articles (n=383) with 5 artificial intelligence tools.

The results of sentiment classification performance are presented as proportion (%) of agreement with the human rater for each tool and sentiment category (Figures2  3). The performance of the AI tools was generally low and varied across sentiment categories and data sources. Agreement was highest for positive sentiment and lowest for neutral sentiment across both platforms. In the GA dataset, SentimentQDAP had the highest agreement with the human rater for negative sentiment (n=28, 62.2%), while SentimentGI had the highest agreement for positive sentiment (n=36, 62.1%), and VADER performed best for neutral sentiment in this dataset, with an agreement rate of 42.9% (n=120). In the X dataset, VADER achieved the highest agreement for negative sentiment (n=78, 54.9%), while ChatGPT-4 demonstrated the highest agreement for both neutral (n=48, 38.2%) and positive (n=92, 55.1%) sentiments. An interrater reliability analysis was performed between the dependent samples of reviewer 1 and each AI tool. For this purpose, Cohen κ was calculated, and in all cases, no agreement was found (Table S3 in Multimedia Appendix 1).

**Figure 3.**
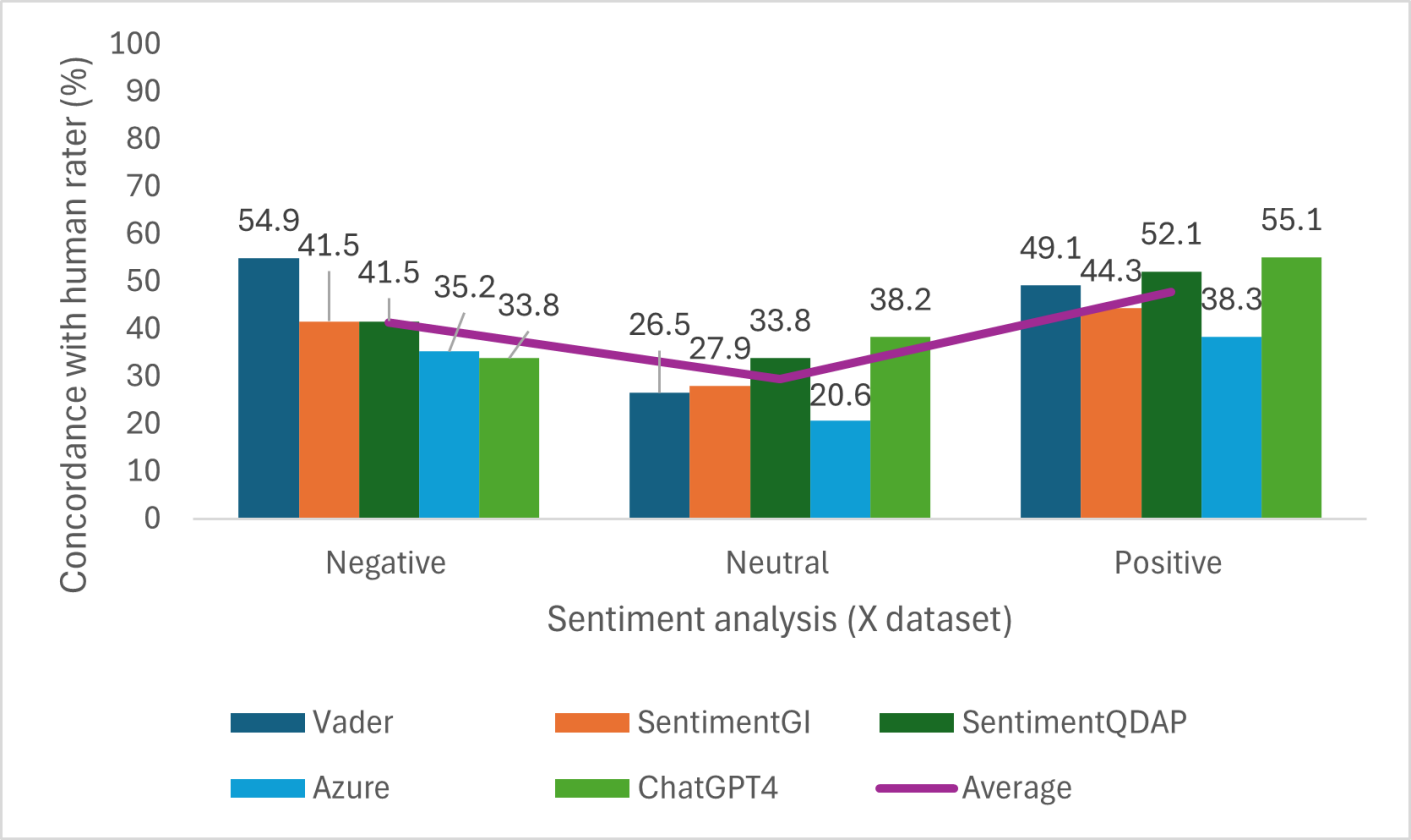
Comparison of human-rated sentiment score for a subset of tweets (n=377) with 5 artificial intelligence tools.

### Manual Sentiment Analysis of Entire Datasets

### Total Sentiments Across the Datasets

Positive, negative, and neutral sentiment results were collated (Table 3), with differences in the distribution of sentiment observed between the 2 platforms. Although the relevant datasets comprised 1283 (57.6%) GA articles and 2473 (%) tweets, a higher number of text segments were analyzed due to articles and tweets that referred to multiple PHMs. These were coded and analyzed separately for each PHM to measure sentiment patterns. In the analysis of the GA platform (n=1587), the majority of the sentiment was neutral (n=1021, 64.3%, 95% CI 62%‐66.7%). Negative sentiments accounted for 18.1% (n=287, 95% CI 16.2%‐20%), while positive sentiments accounted for 17.6% (n=279, 95% CI 15.7%‐19.5%). In the analysis of text segments on the X platform (n=3124), sentiment demonstrated a higher degree of polarity, with negative sentiments comprising 40% (n=1248; 95% CI 38.2%‐41.7%) and positive sentiments comprising 39.5% (n=1233; 95% CI 37.8%‐41.2%) of the total. In the X dataset, 20.6% (n=643; 95% CI 19.2%‐22%) of sentiments were neutral. These results indicate fewer opinions and more neutral reporting of events in the GA dataset, while there was a higher degree of both positive and negative emotional expression found within the X dataset.

**Table 3.** Sentiment distribution across platforms in the entire datasets (Google Alerts [GA] and X).

Platform and sentiment	Frequency (n)	Proportion (95% CI)
GA (n=1587)		
Negative	287	18.1% (16.2%‐20%)
Neutral	1021	64.3% (62%‐66.7%)
Positive	279	17.6% (15.7%‐19.5%)
X (n=3124)		
Negative	1248	40% (38.2%‐41.7%)
Neutral	643	20.6% (19.2%‐22%)
Positive	1233	39.5% (37.8%‐41.2%)

### Sentiment Analysis of Each Search Term

Sentiment analysis results were collated for each search term in each dataset (Table S4 in Multimedia Appendix 1) to investigate sentiment expressed toward specific topics and specific PHM. As described earlier, the various PHMs were discussed in different tones across the 2 platforms, with GA results more likely to be classified as neutral sentiment, and X results more likely to show polarity for each search term. Neutral GA sentiments were expected due to the reporting of events in the news media. For search terms relating to pharmaceutical companies in the X dataset, reports on the search term “Moderna” (n=30), sentiment was more evenly distributed (negative: n=11, 36.7%; neutral: n=10, 33.3%; and positive: n=9, 30%) when compared to sentiment for the search term “Pfizer” (n=222), which was mostly negative (n=135, 60.8%), 30.6% neutral (n=68), and 8.6% positive (n=19). When comparing the search term “booster” (n=187) in X dataset, sentiment was broken down as 40.1% (n=75) negative, 16% (n=30) neutral, and 43.9% (n=82) positive; however, sentiment for the search term “vaccine” (n=860) was mostly negative (n=378, 44%), 28.4% neutral (n=244), and 27.7% positive (n=238). Search terms relating to lockdown (n=23, 54.8%) and mandates (n=85, 66.4%) were associated with negative sentiment in the X dataset, while isolation was associated with positive sentiment (n=17, 85%).

### Sentiment Analysis of Vaccination and Mask Search Terms

Results for search terms related to COVID-19 vaccination and mask-wearing were combined (Table S5 in Multimedia Appendix 1). Vaccine results were analyzed with and without the inclusion of results of the search term “booster,” as it was shown to have opposing polarity when compared to “vaccine” (Table S5 in Multimedia Appendix 1). Analysis of the combined vaccine search terms continues to show the same pattern of distribution between the 2 datasets, with sentiment in the GA dataset predominantly neutral (65.8%) and lower proportions of negative (18.1%) and positive (16.1%) sentiment. In contrast, the X dataset was more polarized and critical of vaccines, with nearly half (49.5%) of all results expressing negative sentiment and lower levels of neutral (26.9%) and positive (23.6%) sentiment (Table S5 in Multimedia Appendix 1). When booster-related results were included in the analysis, these patterns were retained.

The pattern was markedly different for sentiment analysis results of reports collected via mask-related terms. The GA results were also mostly neutral (61.4%), and 26.4% expressing positive sentiment and only 12.3% expressing negative sentiment. In the X dataset, there was a much higher proportion of positive sentiment (66.8%) than negative (21.5%) or neutral (11.7%) sentiments expressed.

### Topic Summary Analysis

LDA topic modeling of the GA and X datasets (Tables S6 and S7 in Multimedia Appendix 1) and the LLM results were compared to the human rating of the top 5 most discussed topics and are presented in Tables4  5, including alignment scores from the cross-matching rubric (Table 2).

**Table 4.** Comparison of human-generated and machine-generated topic analysis of the Google Alerts dataset.

Manually generated topic	RStudio LDA^a^ output	Machine score	LLM^b^ output	LLM score
Prominent physician reveals vaccine injury	Public attitudes toward vaccines and masks	0^c^	Public debate on COVID-19 vaccines	1^d^
China allows travel after lifting of COVID-19 restrictions	COVID-19 mitigation: mask usage and vaccination	1	Mask mandates and usage	1
Impact of vaccine rollout on economy	COVID-19 protection and boosters	1	Economic and social impacts of PHM	2^e^
Easing of COVID-19 restrictions	Australian COVID-19 response	2	COVID-19 vaccine mandates and compliance	1
India—wearing masks due to new COVID-19 wave	Intentions regarding public health measures	0	Global or local responses to COVID-19 variants	2

a LDA: latent Dirichlet allocation.

b LLM: large language model.

c 0: no match.

d 1: partial match.

e 2: direct match.

**Table 5.** Comparison of human-generated and machine-generated topic analysis of X dataset.

Manually generated topic	RStudio LDA^a^ output	Machine score	LLM^b^ output	LLM score
COVID-19 vaccines	Discourse on mask-wearing and COVID-19 vaccination	2^c^	Vaccine safety and side effects	1^d^
New isolation rules—5 days with mask	Individual attitudes toward masks and vaccination	2	Mask usage and effectiveness	2
Wearing masks on a plane	COVID-19 choices including vaccine brands	0^e^	Vaccination campaigns and compliance	2
Wearing masks on public transport	Intention to comply with PHM^f^	1	Public health measures and policy debate	2
China ceases zero-COVID-19 policy	Current perspectives on mask-wearing and vaccination	1	Misinformation and distrust in authorities	0

a LDA: latent Dirichlet allocation.

b LLM: large language model.

c 2: direct match.

d 1: partial match.

e 0: no match.

f PHM: public health measure.

### GA Dataset

Mask use and mask mandates, as well as vaccine mandates, are discussed in the GA dataset within the context of easing COVID-19 restrictions (Table 4). Across the 5 cases, the RStudio LDA output topics fully aligned with 1 of 5 cases and partially aligned with the manual coding in 2 of 5 instances, while the LLM output fully matched in 2 of 5 instances and partially matched in 3 of 5 cases. The LLM outputs show a deeper understanding of the context but are often too generalized, while some of the machine-generated outputs were related but not specific to the manually generated topics.

### X Dataset

Topics discussed in the X dataset included announcements concerning the easing of restrictions, and opinions both supporting and opposing the dropping of mask mandates and prolonged isolation following a positive COVID-19 test (Table 5). Vaccination campaigns and safety concerns were also discussed. Results from the cross-matching rubric of the X dataset showed that the LLM output more closely aligned with the manually generated topic, fully matching in 3 of 5 cases and partially matching in 1 of 5 cases. The LDA output fully matched in 2 of 5 instances and partially matched in 2 of 5. The LLM output is more descriptive of the topics.

### Human-Coded Thematic Analysis

Thematic analysis was performed on the positive- and negative-assigned GA articles and tweets (Tables S8a-S8d in Multimedia Appendix 1). Many tweets were of the account owners reporting their choice to engage or not engage with particular PHMs (eg, “My wife and I wear masks when we go out unless we are eating or drinking or outside. There is no way I am having that experimental vaccine”). Several other themes emerged and are summarized in Table 6.

**Table 6.** Summary of thematic analysis results by large language model (LLM).

Dataset	LLM summary
Positive GA^a^ dataset	“The thematic analysis reveals a comprehensive approach to managing COVID-19, from vaccination campaigns and mask mandates to broader societal adjustments. Public health measures, supported by clear communication, have been instrumental in mitigating the impact of the pandemic. Emerging variants and evolving immunity continue to challenge and shape policies. Overall, the findings underscore the importance of ongoing vigilance and adaptability in public health.”
Negative GA dataset	“The thematic analysis reveals ongoing tensions surrounding vaccine mandates, including public resistance and concerns over safety. Mask mandates evoke polarized opinions, with debates on their necessity and effectiveness. Lockdowns have left lasting societal and psychological impacts, fuelling protests and dissatisfaction. Public health messaging faces challenges due to misinformation and varying adherence levels. The emergence of new COVID-19 variants highlights the need for adaptive vaccination strategies. These findings underscore the complex interplay of public health policies, societal reactions, and individual behaviours.”
Positive X dataset	“The thematic analysis highlights an ongoing tension between public health policies and individual behaviours. Masks and vaccines are central to the discourse, with significant debate over their adoption and effectiveness. Variants like Omicron continue to influence strategies, while community responsibility emerges as a key element in combating the pandemic. These findings underline the complexity of managing public health in a rapidly evolving situation.”
Negative X dataset	“The thematic analysis reveals significant public resistance and scepticism toward COVID-19 measures. Vaccine safety and mask efficacy are hotly debated, often linked with broader concerns about personal freedoms and ethical mandates. Misinformation and a lack of trust in authorities further exacerbate these issues. Public fatigue with ongoing health measures indicates a need for transparent communication and adaptive strategies moving forward.”

a GA: Google Alerts.

In the positive GA dataset, themes included masks being recommended in particular contexts (eg, *“*...masks are recommended in health care settings, on public transport, in crowded indoor settings”) and encouraging vaccination in at-risk groups (eg, “Aside from the government and GPs, family members have an important role in encouraging senior citizens to get vaccines or booster shots against COVID-19”).

In the negative GA dataset, themes included reports of the COVID-19 vaccines causing injuries (eg, “Dr [name] has broken her silence about a ‘devastating’ COVID vaccine injury, slamming regulators for ‘censoring’ public discussion, and Thousands of Australians suffering from COVID-19 vaccine injury feel they are ‘not being heard’ or treated fairly by the government”). Occupational vaccine mandates were also discussed with negative sentiment expressed (eg, “Over 200 firefighters in New South Wales and Victoria are being forced to ‘stay away from saving lives’ because of ongoing vaccine mandates, and Coles is the only major supermarket in Australia that continues to use discriminatory COVID vaccination mandates for workers”).

In the positive X dataset, these included masks being protective for the wearer, with reasons of vulnerability or framing it as “smart” behavior (eg, “My daughter works with COVID patients and we wear a mask when she visits. My partner has cancer, and Lots of people coughing on the train and I’m the only one smart enough to wear a mask”). People also discussed the wearing of masks to protect others in the community (eg, “It is selfish not to wear a mask to protect the health of our most vulnerable”). There were many posts expressing the opinion that the benefits of vaccines outweigh the risks (eg, “I’m sorry for people injured by the vaccine but the risk is insignificant compared to complications from Covid”). When mentioning boosters, the sentiment tended to be favorable, wanting access to updated boosters (eg, “I saw on the news that we might get 5th jab in February – can’t come soon enough!”).

In the negative X dataset, a strong theme emerged about COVID-19 vaccines causing injuries and deaths. Many tweets included statistics regarding vaccine deaths and anecdotal reports of people they know or had heard of dying suddenly after being vaccinated, or reporting lived experience of having a vaccine injury (eg, “Vaccine injury and deaths outnumber actual deaths FROM Covid,” and “My neighbour told me that her son’s friend went down to the local shopping center to get vax, dies 15 minutes later”). There was also a theme that COVID-19 vaccines are experimental, and people who take the vaccine are “brainwashed” and “sheep,” positioning those who had not had the vaccine as “smart” (eg, “Scientists came up with this vaccine in 10 weeks, and people still believe it’s safe, talk about being brainwashed, and The sheep are rolling up their sleeves for their 5th jab, and I don’t inject poison into my body, unlike the vax junkies”).

### Thematic Analysis Results From LLM

The LLM-generated summaries of the thematic analysis of the positive- and negative-assigned GA and X datasets are presented in Tables 7-10. Results of LLM thematic analysis, including theme descriptions and the proficiency and reasonableness scores benchmarked against human-coded thematic analysis, are presented in Table 2. When compared to the human-coded thematic analysis, the results for the LLM proficiency were 13 of 20 proficient and 7 of 20 partially proficient, showing that the LLM produced themes that were relevant to the dataset and closely matched with the human-coded themes. All themes, whether fully or partially proficient at matching human-generated themes, were rated “very reasonable” (the themes had high relevance to the dataset and would likely be generated by human coding), suggesting that the results may be useful to support human coding of large datasets.

**Table 7.** Results of the large language model (LLM)–coded thematic analysis of positive and negative Google Alert (GA) results and tweets: themes from the positive GA dataset.

Theme	Description	Example of a representative GA result	Proficiency score^a^	Reasonableness score^b^
COVID-19 vaccination and immunity	Emphasis on vaccination rollouts, booster campaigns, and their impact on immunity Subthemes: Effectiveness and benefits: vaccines reducing ICU^c^ admissions and mortality Booster campaigns: urging booster shots to combat waning immunity Vaccination messaging: efforts to dispel misinformation and encourage uptake	“Vaccination against COVID-19 disease aims to reduce the severity of symptoms and need for hospitalizations.” “A booster dose can safeguard against severe illness.” “Vaccination was also found to be a cost-saving measure.”	Proficient	2
Mask-wearing policies	Role of mask mandates in mitigating viral spread Subthemes: Mandates and compliance: discussion on mandatory versus voluntary mask use Context-specific recommendations: masks encouraged in health care and public transport	“Masks should be compulsory this Christmas to quash a fresh spike in Covid cases.” “Masks are recommended in healthcare settings and crowded indoor spaces.”	Proficient	2
Economic and social effects	Evaluation of public health measures on economic stability and daily life Subthemes: Economic savings: prevention of losses via vaccination and lockdown measures Social changes: adaptations during and after pandemic	“The vaccine rollout saved the national economy $181bn in potential damage.” “Australia’s hospitality venues are recording a boom in revenue as we enjoy post-Covid freedoms.”	Proficient	2
Emerging variants and evolving strategies	Addressing new COVID-19 strains and their implications Subthemes: Variant-specific concerns: Omicron and immunity challenges Hybrid immunity: interplay of natural and vaccine-induced immunity	“Countries should consider recommending masks on long-haul flights given the spread of Omicron.” “Hybrid immunity has contributed to poor vaccine booster uptake.”	Proficient (LLM explores variants and immunity more comprehensively than human coding)	2
Public health communication	Messaging around guidelines and behavioral changes Subthemes: Hygiene and ventilation: promoting preventive measures Community engagement: tailored campaigns for vulnerable populations	“Good ventilation assists in reducing the risk of spreading airborne diseases.” “Health experts are warning Australians not to miss important screenings and vaccinations.”	Proficient (LLM explores messaging around all PHMs^d^ more comprehensively than human coding)	2

a Proficiency score: proficient, partially proficient, not proficient. and not proficient.

b Reasonableness score: 2=very reasonable, 1=reasonable, and 0=not reasonable.

c ICU: intensive care unit.

d PHM: public health measure.

**Table 8.** Results of the large language model (LLM)–coded thematic analysis of positive and negative Google Alert (GA) results and tweets: themes from the negative GA dataset.

Theme	Description	Example of a representative GA result	Proficiency score	Reasonableness score
Vaccine mandates and resistance	Explores public reactions to vaccine mandates, including compliance and opposition Subthemes: Compliance challenges: public debates on mandates for essential workers and specific industries Resistance and injuries: reports of adverse reactions and public resistance	“Coles is the only major supermarket in Australia that continues to employ discriminatory Covid vaccination mandates for workers.” “Dr (name) has spoken out about her wife’s ‘severe’ reaction to the COVID jab.”	Proficient	2
Mask mandates	Discussion on the effectiveness, compliance, and psychological effects of mask-wearing Subthemes: Effectiveness and compliance: questioning the effectiveness of mask mandates Public perception: mixed reactions to prolonged mask-wearing requirements	“More embarrassing for Team Mask, those even more uncomfortable N95 masks made little to no difference either.” “On lone mopeds, driving their own cars, on goes the mask.”	Proficient	2
Lockdowns and societal effects	Highlights the societal consequences of lockdowns, including protests and mental health issues Subthemes: Protests and backlash: resistance to prolonged lockdowns Mental health impacts: isolation and its effects on well-being	“Fairfield and other areas of western Sydney were subject to strict COVID-19 lockdowns.” “Most young Australians have come away feeling lonely and left out.”	Proficient	2
Public health messaging	Examines the effectiveness of communication around health measures Subthemes: Misinformation and clarity: confusion around vaccine safety and mandates Behavioral recommendations: promoting hygiene and mask use	“Covid frightbat GP previously railed against vaccine misinformation that scared people away from being vaccinated.” “Masks are no longer suggested in public spaces.”	Proficient (LLM explores messaging around PHMs more comprehensively than human coding)	2
Emerging variants and vaccination strategies	Discusses adaptive responses to new COVID-19 variants Subthemes: Variant-specific challenges: addressing immunity gaps Booster campaigns: advocacy for continued vaccination efforts	“Waning immunity is apparent amongst many communities.” “Booster doses continue to help the most vulnerable even as more contagious variants have popped up.”	Proficient (LLM explores variant-specific issues and immunity more comprehensively than human coding)	2

a Proficiency score: proficient, partially proficient, not proficient. and not proficient.

b Reasonableness score: 2=very reasonable, 1=reasonable, and 0=not reasonable.

**Table 9.** Results of the large language model (LLM)–coded thematic analysis of positive and negative Google Alert (GA) results and tweets: themes from positive X dataset.

Theme	Description	Example of a representative tweet	Proficiency score^a^	Reasonableness score^b^
Mask usage and debate	Conversations about the use of masks as a public health tool Subthemes: Mandatory masking: debates over government-mandated mask policies Efficacy and preferences: discussions on the effectiveness of different types of masks (eg, N95 vs cloth) Public compliance: observations on varying levels of mask adoption	“Mandate masks on planes would be the bare minimum.” “N95 masks are your best line of defence. Take care.” “Flew from Newcastle to Brisbane today. Apart from us, only one other family wore masks.”	Proficient	2
Vaccination and public perception	Focuses on vaccination campaigns, uptake, and public attitudes Subthemes: Booster campaigns: efforts to encourage additional doses for improved immunity Safety and side effects: concerns about vaccine side effects and injuries Resistance and advocacy: voices both supporting and opposing vaccination	“The public must get the 4th vax and mask up.” “COVID vaccines have caused 14 deaths. These deaths are tragic but were expected.” “Vax works!! Vaccines are definitely a better protection than masks.”	Partially proficient (human coding also focused on the benefits of vaccines and effectiveness)	2
Policy and public health strategies	Explores government policies and public discourse on their effectiveness Subthemes: Lockdown measures: reflections on the impact and necessity of lockdowns Social distancing and hygiene: recommendations for maintaining distance and promoting hygiene	“We needed a nuanced COVID-19 policy that included indoor masks.” “Masks and social distancing work. It was a good run while it lasted.”	Proficient	2
Emerging COVID-19 variants	Discusses the impact of new COVID-19 variants on public health responses Subthemes: Omicron and immunity: challenges posed by the Omicron variant Evolving strategies: adjusting public health measures in response to new variants	“Waning immunity is apparent amongst many communities.” “Variants mean changing our habits.”	Partially proficient (human coding also focused on wanting access to boosters due to new variants, and PHM generally)	2
Community behavior and responsibility	Focus on individual and collective adherence to health measures Subthemes: Risk awareness: acknowledging personal and societal risks Public responsibility: encouragement for collective action to mitigate risks	“We wear masks to save others’ lives as well.” “Masks are our only weapon against COVID. This method has worked for us so far.”	Proficient	2

a Proficiency score: proficient, partially proficient, and not proficient.

b Reasonableness score: 2=very reasonable, 1=reasonable, and 0=not reasonable.

**Table 10.** Results of the large language model (LLM)–coded thematic analysis of positive and negative Google Alert (GA) results and tweets: themes from the negative X dataset.

Theme	Description	Example of a representative tweet	Proficiency score^a^	Reasonableness score^b^
Vaccine safety and side effects	Concerns about vaccine-induced side effects dominate the conversation Subthemes: Personal accounts of side effects: tweets describing adverse events Skepticism toward safety testing: critiques of vaccine trial processes Call for accountability: demands for recognition and compensation for injuries	“My 24-year-old nephew collapsed after the Pfizer vaccine and now suffers from myocarditis.” “Emergency vaccines weren’t tested long enough for safety.” “The government must acknowledge vaccine injuries and help those affected.”	Proficient	2
Mask effectiveness and resistance	Heated debate on the effectiveness and necessity of masks Subthemes: Efficacy questions: disputes over the scientific basis for masks Resistance to mandates: opposition to mask-wearing policies Cultural and behavioral aspects: comments on individual mask-wearing habits	“Masks don’t work to reduce transmission at a population level.” “Stop forcing masks on us. We deserve freedom of choice.” “People wearing masks alone in their cars – what’s the point?”	Proficient	2
Economic and social misinformation and distrust	Mistrust in authorities and allegations of misinformation Subthemes: Media and government distrust: criticism of public health messaging Manipulation allegations: claims of hidden agendas behind health measures Need for transparency: calls for open sharing of data and studies	“Mainstream media keeps spreading vaccine propaganda.” “The pandemic was planned to control us through fear and mandates.” “We need real data on vaccine injuries, not censored narratives.”	Partially proficient (human coding also focused on the role of pharmaceutical companies, not just media and government)	2
Ethical concerns about mandates	Ethical debates around the imposition of health mandates Subthemes: Coercion and freedom: mandates seen as violations of personal liberty Social divisions: how mandates create tension and polarization Impact on vulnerable groups: concerns about marginalized communities	“No one should be forced to take a vaccine against their will.” “Stop dividing us into vaxxed and unvaxxed groups.” “Mandates disproportionately affect those with medical exemptions.”	Partially proficient (human coding also focused on the stereotyping of people following government advice as brainwashed)	2
Long-term public health strategies	Critical evaluation of measures like boosters and lockdowns Subthemes: Effectiveness of boosters: mixed reactions to additional doses Policy fatigue: frustration with prolonged measures Variant-specific adaptations: adjustments in strategy due to new variants	“How many boosters are we expected to take? It’s exhausting.” “Lockdowns destroyed businesses and mental health.” “Omicron shows we need better vaccines, not just boosters.”	Proficient	2

a Proficiency score: proficient, partially proficient, not proficient. and not proficient.

b Reasonableness score, 2=very reasonable, 1=reasonable, 0=not reasonable.

## Discussion

### Principal Findings

Our study compared the results of AI analysis of PHM-related datasets with human-coded analysis for common tasks used in the context of health emergencies, such as sentiment analysis (to explore public opinion of PHMs), topic modeling (to identify what is being discussed in online news and social media), and thematic analysis (for a more in-depth analysis of how PHMs are being discussed in the public domain). AI tools were deliberately selected to reflect those accessible to public health professionals in Australia that require minimal technical expertise.

Overall, AI tools performed inconsistently across tasks. All models showed poor performance for sentiment analysis. ChatGPT-4 was found to demonstrate stronger alignment with human raters for both the topic modeling and thematic analysis tasks. These findings highlight both the potential and the limitations of AI tools to complement traditional methods of analysis for public health professionals by providing rapid insights while still requiring human interpretation and oversight [26].

### Sentiment Analysis Task

To evaluate the accuracy of accessible AI sentiment analysis tools, a subset of Australian English-language GA and X datasets was analyzed by 2 human raters and 5 AI tools. Sentiment was poorly detected by all AI tools, with no agreement between the human rater and either the GA or X datasets (Table 3). In particular, accuracy was less than 30% for neutral sentiment in the GA dataset and under 50% for positive or negative sentiment in the X dataset. These findings align with previous studies showing that off-the-shelf sentiment analysis tools perform poorly when applied to complex, health-related discourse [54,55].

The human-coded sentiment analysis showed that the GA dataset had a majority of neutral sentiment in discussion about PHMs, while the X dataset was evenly split between positive and negative. These findings highlight differences between the data sources, with the prevalence of neutral sentiment in the GA dataset, suggesting predominantly neutral reporting of current stories and events in online news media. The X dataset showed greater polarity of sentiment, which may indicate that users of X have stronger opinions and are more likely to express them online. Our study demonstrates that in Australia during the study period, mask use and isolation were associated with more positive sentiment, while vaccines, lockdowns, and mandates attracted more negativity. These results indicate that sentiment analysis may give useful high-level insights regarding public opinion for public health decision-makers while highlighting the need for contextual interpretation by human analysts.

### Topic Analysis Task

In our study, topic modeling identified overlapping areas of discussion between datasets, with GA focusing on mask use and vaccine mandates in the context of easing restrictions and X focusing on vaccination campaigns, mask mandates, and isolation requirements. LDA partially aligned with human-coded topics, while the LLM output provided fuller contextualization but was overly generalized. Recent advances in generative AI have shown improved topic matching with human annotators of health-related datasets using LLMs [52]. This may assist in public health responses during health emergencies by improving the understanding of topics that are being discussed in the news and on social media and addressing misunderstandings or concerns with public health messaging [56].

### Thematic Analysis Task

Qualitative research methods are promoted as the most suitable approach to gain an understanding of the experiences of individuals during health emergencies, which can be used to inform local public health policy decisions and implementation [10]. Manual interpretation of data, including thematic analyses, presents an extensive time and resource burden [52]. Generative LLMs can analyze and interpret vast amounts of text and have shown good accuracy in generating themes when compared to human analysts, with adequate depth of explanations of themes and inclusion of appropriate quotations, with time savings of several hours or even days [13,52,57]. Some studies have suggested that thematic analysis results generated by LLMs may be best used in collaboration with human coders with domain-specific knowledge [14,58,59].

The human-coded thematic analysis of the GA dataset (Table S8a-d in Multimedia Appendix 1) revealed the ways in which PHMs were being discussed in the news media, including strong support for vaccination of at-risk groups and the use of masks in specific high-transmission contexts, while also reporting on vaccine injuries. Themes that emerged from the X dataset revealed polarized views on the uptake of PHM, with the positioning of compliance with vaccines and mask-wearing as “smart” by supporters of those PHM. From the opposing view, there was much discussion about vaccine harms, including injuries and deaths, and the positioning of compliance with PHMs as a result of “brainwashing.” There were also reports of sudden death from the “experimental vaccine,” which is useful for public health professionals to be aware of when planning campaigns to combat misinformation [60].

In our study, the LLM-generated themes were relevant to the dataset, and the majority were closely matched with the human-coded themes. Even when the themes were only partially matched, they rated high on how reasonable it was for the LLM to generate the theme from the dataset, suggesting that thematic analysis may provide valuable input to inform public health decision-making in a timely way. The summaries generated by the LLM provided an overview of the AI-generated thematic analysis; however, they lacked the specific insights and understanding of the social context of the human-generated summaries.

### Error and Bias Analysis

Errors in sentiment classification and theme generation were consistent across tools and datasets in this study, reflecting known limitations of NLP and LLM models when applied to public health discourse [55,61,62]. Misclassification of neutral sentiment was frequent, which may result from narrow sentiment thresholds and training data that are not designed for health-related news and social media [54]. Inability to detect sarcasm is another known limitation of AI tools for sentiment and thematic analysis tasks, when the textual data include positive words to express negative sentiment, highlighting another area where human oversight for contextual nuance is important for accurate analysis [63,64]. While LLMs have demonstrated superior accuracy over other AI tools for tasks such as sentiment analysis, topic modeling, and thematic analysis of public health datasets, they do not match human raters for interpretation and depth of analysis [13,52,55,65]. Fine-tuned or domain-specific LLMs trained on health-related text may improve accuracy, but these models are underrepresented, and substantial annotation and validation efforts will be required to develop appropriately trained models for public health contexts [63-65].

LLM reproducibility presents another challenge, as outputs may vary over time as model parameters and training data are updated by developers. This is known as “model drift” and may complicate longitudinal comparisons and replication of results [66]. Researchers should record model versions and exact prompt wording (as in Table S1 in Multimedia Appendix 1) to enhance transparency and allow future verification.

Manual analysis of online public opinion data may be open to bias through the interpretation of the researcher [67]; however, automated methods lack the capacity to clarify the results of analysis, as may be possible with more traditional methods [66]. While social media can give voice to more marginalized groups, health inequities can be amplified if unrepresentative data are used for analysis [68]. Social bots, which are computer algorithms designed to mimic human interactions on social media, can be used to manipulate public opinion and therefore skew sentiment data [69].

### Limitations

There were several limitations of our study. First, regarding the data sources used, GA retrieved the first 2 sentences of the article, which may not give an accurate representation of the sentiment of the article. While the anonymity of data from online social networking sites may have benefits over qualitative or survey data by reducing the impact of social desirability bias [70,71], the awareness of the post being observed by others may make the user less likely to publish unpopular opinions [72]. The anonymity of social media data also impacts the ability to collect demographic information, which can impact how generalizable the results of the analysis [73]. The use of social media data for research is becoming increasingly challenging due to restrictions on access by commercial owners of the platform [74]. The use of untrained NLP sentiment analysis tools, which were unable to correctly identify both neutral sentiment and sarcasm, was a further limitation of this study. While AI can offer a timely way to provide real-time data, public health professionals may be skeptical of the results without understanding the process and how to interpret the output; therefore, education on its effective use will be necessary for future implementation [75]. Inequities and bias that are present in the training data may be replicated in AI outputs [75]; for example, LLMs trained primarily on content originating from North America and the United Kingdom may misrepresent Australian cultural and linguistic nuances, potentially skewing thematic outputs in subtle ways [75]. As this study was conducted on an English-language dataset, the results of this analysis do not capture sentiment and thematic perspectives from culturally and linguistically diverse communities [1]. This limitation is particularly important in multicultural settings, such as Australia, where attitudes toward PHMs may differ across language groups [1]. For AI techniques to inform public health policies in Australia, they must support an understanding of the actual sentiment in diverse communities. While automatic translation and multilingual models can be used for analysis of text in multiple languages, there is a dearth of available data for analysis in languages other than English in the Australian context.

### Conclusions

During health emergencies, there is a need to balance rapid analysis of data with accuracy to support public health decision-making. This study examined the accuracy of 5 AI tools in performing tasks designed to measure public opinion in Australia toward PHMs, such as vaccines, mask mandates, and lockdowns. AI tools were chosen that are widely available across government agencies in Australia. All AI tools were found to perform poorly in a sentiment analysis task of the GA and X datasets when compared to a human rater. AI-generated topic modeling and thematic analysis were conducted using the LLM ChatGPT-4 and compared to human-generated responses. The LLM topic modeling outputs showed a high level of alignment with the human-generated topics, and while understanding of the context of the results was indicated, it was often overly generalized. The LLM output of the thematic analysis task was found to be highly relevant and well matched to the human-generated analysis. Even when the themes were only partially matched to the human-generated themes, the results were classified as reasonable, relevant to the data, and likely to be generated by a human analyst. These findings suggest that AI tools, particularly LLMs, may serve as a rapid triage tool to surface emergent themes from large-scale public datasets, which could then be reviewed or refined by human analysts in time-sensitive policy settings. It is unlikely that AI tools will replace traditional research methods used to investigate attitudes and opinions to epidemic PHMs in Australia; however, there remains an opportunity to use this technology to complement qualitative research techniques used by public health professionals in a cost-effective and timely way in the context of health emergencies.

## Supplementary material

10.2196/80824 Multimedia Appendix 1 Additional tables.

10.2196/80824 Multimedia Appendix 2 Code for latent Dirichlet allocation.

## Abbreviations

AI: artificial intelligence
API: application programming interface
GA: Google Alerts
LDA: latent Dirichlet allocation
LLM: large language model
NLP: natural language processing
PHM: public health measure
VADER: Valence Aware Dictionary and Sentiment Reasoner

## References

[1] Jacobs LR, Mettler S (2011). Why public opinion changes: the implications for health and health policy. J Health Polit Policy Law.

[2] Tahamtan I, Potnis D, Mohammadi E, Singh V, Miller LE (2022). The mutual influence of the World Health Organization (WHO) and Twitter users during COVID-19: network agenda-setting analysis. J Med Internet Res.

[3] Yu G, Garee M, Ventresca M, Yih Y (2024). How individuals’ opinions influence society’s resistance to epidemics: an agent-based model approach. BMC Public Health.

[4] (2024). Disinformation and public health. World Health Organization.

[5] Denniss E, Lindberg R (2025). Social media and the spread of misinformation: infectious and a threat to public health. Health Promot Int.

[6] Nutbeam D, Milat AJ (2025). Artificial intelligence and public health: prospects, hype and challenges. Public Health Res Pract.

[7] Alcantara AM, Saeri A (2024). Insights to action: an analysis of the COVID-19 pulse survey. https://anzsog.edu.au/insights/insights-to-action-an-analysis-of-the-covid-19-pulse-survey.

[8] Vindrola-Padros C, Chisnall G, Cooper S (2020). Carrying out rapid qualitative research during a pandemic: emerging lessons from COVID-19. Qual Health Res.

[9] (2022). Rapid qualitative research to increase COVID-19 vaccination uptake: a research and intervention tool. World Health Organization.

[10] Tremblay S, Castiglione S, Audet LA, Desmarais M, Horace M, Peláez S (2021). Conducting qualitative research to respond to COVID-19 challenges: reflections for the present and beyond. Int J Qual Methods.

[11] Taylor B, Henshall C, Kenyon S, Litchfield I, Greenfield S (2018). Can rapid approaches to qualitative analysis deliver timely, valid findings to clinical leaders? A mixed methods study comparing rapid and thematic analysis. BMJ Open.

[12] Dong D, Abramowitz S, Matta GC (2023). A rapid qualitative methods assessment and reporting tool for epidemic response as the outcome of a rapid review and expert consultation. PLOS Glob Public Health.

[13] Prescott MR, Yeager S, Ham L (2024). Comparing the efficacy and efficiency of human and generative AI: qualitative thematic analyses. JMIR AI.

[14] Lancaster K, Rhodes T, Rosengarten M (2020). Making evidence and policy in public health emergencies: lessons from COVID-19 for adaptive evidence-making and intervention. Evid Policy.

[15] Wang C, Wang X, Wang P, Deng Q, Liu Y, Zhang H (2024). Evaluating public opinions: informing public health policy adaptations in China amid the COVID-19 pandemic. Sci Rep.

[16] Han X, Wang J, Zhang M, Wang X (2020). Using social media to mine and analyze public opinion related to COVID-19 in China. IJERPH.

[17] Hu T, Wang S, Luo W (2021). Revealing public opinion towards COVID-19 vaccines with Twitter data in the United States: spatiotemporal perspective. J Med Internet Res.

[18] Mavragani A, Gkillas K (2020). COVID-19 predictability in the United States using Google Trends time series. Sci Rep.

[19] Venkatesh U, Gandhi PA (2020). Prediction of COVID-19 outbreaks using Google Trends in India: a retrospective analysis. Healthc Inform Res.

[20] Leeson W, Resnick A, Alexander D, Rovers J (2019). Natural language processing (NLP) in qualitative public health research: a proof of concept study. Int J Qual Methods.

[21] Wang Z, Ma Y, Song Y, Huang Y, Liang G, Zhong X (2024). The utilization of natural language processing for analyzing social media data in nursing research: a scoping review. J Nurs Manag.

[22] Lyu JC, Han EL, Luli GK (2021). COVID-19 vaccine-related discussion on Twitter: topic modeling and sentiment analysis. J Med Internet Res.

[23] Yousef M, Dietrich T, Rundle-Thiele S (2022). Actions speak louder than words: sentiment and topic analysis of COVID-19 vaccination on Twitter and vaccine uptake. JMIR Form Res.

[24] White BK, Wilhelm E, Ishizumi A (2024). Informing social media analysis for public health: a cross-sectional survey of professionals. Arch Public Health.

[25] Bragge P, Kellner P, Tsering D, Delafosse V (2024). Use of public opinion data to inform COVID-19 policymaking. ANZSOG.

[26] Mavragani A (2020). Infodemiology and infoveillance: scoping review. J Med Internet Res.

[27] Mallawaarachchi V, Meegahapola L, Madhushanka R, Heshan E, Meedeniya D, Jayarathna S (2021). Change detection and notification of web pages. ACM Comput Surv.

[28] Google Alerts. Google.

[29] Kelly P (2023). Lessons from the fourth Omicron COVID-19 wave. Advice from the Chief Medical Officer, Professor Paul Kelly [press release]. https://www.health.gov.au/sites/default/files/2023-03/lessons-from-the-fourth-omicron-covid-19-wave-chief-medical-officer-professor-paul-kelly.pdf.

[30] Bour C, Ahne A, Schmitz S, Perchoux C, Dessenne C, Fagherazzi G (2021). The use of social media for health research purposes: scoping review. J Med Internet Res.

[31] (2024). About different types of posts. X Help Center.

[32] (2023). Microsoft Released Update v2311 (build 17029.20108 c2r) current channel for Microsoft Office 365 Products on 12th December 2023. Microsoft Build 2026.

[33] McHugh ML (2012). Interrater reliability: the kappa statistic. Biochem Med (Zagreb).

[34] van Atteveldt W, van der Velden M, Boukes M (2021). The validity of sentiment analysis: comparing manual annotation, crowd-coding, dictionary approaches, and machine learning algorithms. Commun Methods Meas.

[35] Joshi KP, Jamadar DC (2021). Statistical software applications and statistical methods used in community medicine and public health research studies. Natl J Community Med.

[36] Bertke SJ, Kelly-Reif K (2022). Introducing LTASR, a new R package based on the NIOSH Life Table Analysis System. Occup Environ Med.

[37] Heinsberg LW, Koleck TA, Ray M, Weeks DE, Conley YP (2023). Advancing nursing research through interactive data visualization with R Shiny. Biol Res Nurs.

[38] Digital Transformation Agency (2024). Australian Government Trial of Microsoft 365 Copilot. Australian Government.

[39] (2023). Azure AI apps and agents. Microsoft.

[40] Hutto CJ, Gilbert E VADER: a parsimonious rule-based model for sentiment analysis of social media text. ICWSM.

[41] Feuerriegel S, Pröllochs N (2021). SentimentAnalysis: dictionary-based sentiment analysis. CRAN Project.

[42] Achiam J, Adler S, Agarwal S, Ahmad L, Akkaya I, OpenAI (2023). GPT-4 technical report. https://cdn.openai.com/papers/gpt-4.pdf.

[43] Roehrick K (2022). CRAN: Package “vader”. https://cran.r-project.org/web/packages/vader/vader.pdf.

[44] Feuerriegel S, Pröllochs N (2023). CRAN: package “sentimentanalysis”. https://github.com/sfeuerriegel/SentimentAnalysis.

[45] (2024). Tutorial: text analytics with Azure AI services. Microsoft.

[46] (2025). ChatGPT (January 2025 version). OpenAI.

[47] Braun V, Clarke V (2022). Thematic Analysis: A Practical Guide.

[48] (2023). R Foundation for Statistical Computing.

[49] Wittmann FH (2024). Enhancing thematic analysis with large language models: a comparative study of structured prompting techniques. https://www.diva-portal.org/smash/get/diva2:1939104/FULLTEXT02.

[50] Braun V, Clarke V (2006). Using thematic analysis in psychology. Qual Res Psychol.

[51] Deiner MS, Honcharov V, Li J, Mackey TK, Porco TC, Sarkar U (2024). Large language models can enable inductive thematic analysis of a social media corpus in a single prompt: human validation study. JMIR Infodemiology.

[52] Allaire JJ (2020). RStudio: integrated development environment for for R. https://www.r-project.org/conferences/useR-2011/abstracts/180111-allairejj.pdf.

[53] He L, Omranian S, McRoy S, Zheng K Using large language models for sentiment analysis of health-related social media data: empirical evaluation and practical tips. medRxiv.

[54] Lossio-Ventura JA, Weger R, Lee AY (2024). A comparison of ChatGPT and fine-tuned open pre-trained transformers (OPT) against widely used sentiment analysis tools: sentiment analysis of COVID-19 survey data. JMIR Ment Health.

[55] Chipidza W, Akbaripourdibazar E, Gwanzura T, Gatto NM (2022). Topic analysis of traditional and social media news coverage of the early COVID-19 pandemic and implications for public health communication. Disaster Med Public Health Prep.

[56] Yang Y, Alba C, Wang C, Wang X, Anderson J, An R (2024). GPT models can perform thematic analysis in public health studies, akin to qualitative researchers. J Soc Comput.

[57] Nguyen-Trung K (2025). ChatGPT in thematic analysis: can AI become a research assistant in qualitative research?. Qual Quant.

[58] Liu A, Sun M (2025). From voices to validity: leveraging large language models (LLMs) for textual analysis of policy stakeholder interviews. AERA Open.

[59] Muric G, Wu Y, Ferrara E (2021). COVID-19 vaccine hesitancy on social media: building a public Twitter data set of antivaccine content, vaccine misinformation, and conspiracies. JMIR Public Health Surveill.

[60] Villanueva-Miranda I, Xie Y, Xiao G (2025). Sentiment analysis in public health: a systematic review of the current state, challenges, and future directions. Front Public Health.

[61] He L, Yin T, Zheng K (2022). They May Not Work! An evaluation of eleven sentiment analysis tools on seven social media datasets. J Biomed Inform.

[62] Joshi A, Bhattacharyya P, Carman MJ (2018). Automatic sarcasm detection. ACM Comput Surv.

[63] Tan YY, Chow CO, Kanesan J, Chuah JH, Lim Y (2023). Sentiment analysis and sarcasm detection using deep multi-task learning. Wirel Pers Commun.

[64] Espinosa L, Salathé M (2024). Use of large language models as a scalable approach to understanding public health discourse. PLOS Digit Health.

[65] Giles EL, Adams JM (2015). Capturing public opinion on public health topics: a comparison of experiences from a systematic review, focus group study, and analysis of online, user-generated content. Front Public Health.

[66] Zolnoori M, Huang M, Patten CA (2019). Mining news media for understanding public health concerns. J Clin Transl Sci.

[67] Fisher S, Rosella LC (2022). Priorities for successful use of artificial intelligence by public health organizations: a literature review. BMC Public Health.

[68] Weng Z, Lin A (2022). Public opinion manipulation on social media: social network analysis of Twitter Bots during the COVID-19 pandemic. Int J Environ Res Public Health.

[69] Kanchan S, Gaidhane A (2023). Social media role and its impact on public health: a narrative review. Cureus.

[70] Bispo Júnior JP (2022). Social desirability bias in qualitative health research. Rev Saude Publica.

[71] Olteanu A, Castillo C, Diaz F, Kıcıman E (2019). Social data: biases, methodological pitfalls, and ethical boundaries. Front Big Data.

[72] Mellon J, Prosser C (2017). Twitter and Facebook are not representative of the general population: political attitudes and demographics of British social media users. Research & Politics.

[73] Takahashi M, Bettinson M (2024). Analyzing online public discourse in Australia: Australian Twittersphere and NewsTalk corpora. Australian Journal of Linguistics.

[74] Olawade DB, Wada OJ, David-Olawade AC, Kunonga E, Abaire O, Ling J (2023). Using artificial intelligence to improve public health: a narrative review. Front Public Health.

[75] Wang W, Jiao W, Huang J (2024). Not all countries celebrate thanksgiving: on the cultural dominance in large language models.

