# Supplementary material for: Comparison of Artificial Intelligence Tools With Human Coding for Sentiment, Topic, and Thematic Analysis Tasks of Public Health Datasets During the COVID-19 Pandemic in Australia: Case Study
Source: Online J Public Health Inform. 2026 Apr 7;18:e80824. doi: 10.2196/80824 (PMC13063369; doi:10.2196/80824)
Supplement: Multimedia Appendix 1 [file ojphi-v18-e80824-s001.docx]

**Supplementary files**

**Table S1**

*Generative AI Prompts for Topic Modelling and Thematic Analysis*

|  | **GA prompt** | **X prompt** |
| --- | --- | --- |
| **Topic Analysis** | I have attached a word file. The sentences in this file are Google alerts results of Australian news Dec 2022 to Feb 2023. I would like you to perform a topic analysis of the content of this file. The output I would like is:   1. A description of the methodology you will use to perform the topic analysis 2. A list of the top 5 topics, with descriptive titles assigned to each topic based on the associated key word. 3. A summary of your findings | I have attached a word file. The sentences in this file are results of a search for tweets located in Australia between Dec 2022 to Feb 2023 regarding public health measures. Ensure data preprocessing has included removal of twitter handles, URLs, stop words and punctuation, terms are standardized (e.g., “vaccination” and “vaccine”), and tokenize the text. I would like you to perform a topic analysis of the content of this file. The output I would like is:   1. A description of the methodology you used to perform the topic analysis 2. A list of the top 5 topics, with descriptive titles assigned to each topic based on the associated key word. 3. A summary of your findings |
| **Thematic Analysis** | I have attached a word file. The sentences in this file are Google alerts results of Australian news Dec 2022 to Feb 2023. I would like you to perform a detailed thematic analysis of the content of this file. The output I would like is:   1. A description of the methodology you used to perform the thematic analysis 2. A detailed thematic analysis of the content including themes with summary of content, a description of subthemes and representative sentences for each subtheme. 3. Please explore themes relating to covid-19 and specific public health measures (such as vaccination, mask wearing, boosters, lockdowns, mandates etc.) and any other additional themes which emerge from the data 4. Provide a summary of your thematic analysis findings | I have attached a word file. The sentences in this file are results of a search for tweets located in Australia between Dec 2022 to Feb 2023 regarding public health measures. Ensure data preprocessing has included removal of twitter handles, URLs, stop words and punctuation, terms are standardized (e.g., “vaccination” and “vaccine”), and tokenize the text. I would like you to perform a detailed thematic analysis of the content of this file. The output I would like is:   1. A description of the methodology you used to perform the thematic analysis 2. A detailed thematic analysis of the content including themes with summary of content, a description of subthemes and representative sentences (paraphrased) for each subtheme. 3. Please explore themes relating to covid-19 and specific public health measures (such as vaccination, mask wearing, boosters, lockdowns, mandates etc.) and any other additional themes which emerge from the data 4. Provide a summary of your thematic analysis findings |

**Table S2**

*Concordance calculations for comparison of human rated and machine rated sentiment analysis (proportion (%) of agreement with human rater for each tool and sentiment category)*

| **Tool** | **Negative (%) GA** | **Negative (%) X** | **Neutral (%) GA** | **Neutral (%) X** | **Positive (%) GA** | **Positive (%) X** |
| --- | --- | --- | --- | --- | --- | --- |
| Vader | 55.6 | 54.9 | 42.9 | 26.5 | 56.9 | 49.1 |
| SentimentGI | 46.7 | 41.5 | 24.3 | 27.9 | 62.1 | 44.3 |
| SentimentQDAP | 62.2 | 41.5 | 25.4 | 33.8 | 51.7 | 52.1 |
| Azure | 40 | 35.2 | 23.6 | 20.6 | 56.9 | 38.3 |
| Chat GPT4 | 31.1 | 33.8 | 35 | 38.2 | 58.6 | 55.1 |
| **Average** | **47.8** | **41.4** | **30** | **29.4** | **57.2** | **47.8** |

**Table S3**

*Cohen’s Kappa scores for inter-rater reliability between human-rated sentiment scores for subset of Google alerts (n=383) and tweets (n=377) with 5 machine-based tools*

|  |  | Cohen's Kappa | Level of agreement |
| --- | --- | --- | --- |
| GA results | Vader | 0.18 | None |
|  | SentimentGI | 0.08 | None |
|  | SentimentQDAP | 0.08 | None |
|  | Azure | 0.04 | None |
|  | Chat GPT4 | 0.07 | None |
| X results | Vader | 0.17 | None |
|  | SentimentGI | 0.07 | None |
|  | SentimentQDAP | 0.14 | None |
|  | Azure | -0.03 | None |
|  | ChatGPT4 | 0.13 | None |

**Table S4**

*Human-coded sentiment analysis by search term of entire dataset*

| **Search term** | **Sentiment** | **Frequency** | **Proportion (95% CI)** | **Frequency** | **Proportion (95% CI)** |
| --- | --- | --- | --- | --- | --- |
| **Platform** |  | **GA** | | **X** | |
| **Moderna** | Negative | 9 | 12.7% (4.9% - 20.4%) | 11 | 36.7% (19.4% - 53.9%) |
|  | Neutral | 55 | 77.5% (67.7% - 87.2%) | 10 | 33.3% (16.5% - 50.2%) |
|  | Positive | 7 | 9.9% (2.9% - 16.8%) | 9 | 30.0% (13.6% - 46.4%) |
| Total |  | 71 |  | 30 |  |
|  |  |  |  |  |  |
| **vax** | Negative | 4 | 8.0% (0.5% - 15.5%) | 159 | 46.6% (41.3% - 51.9%) |
|  | Neutral | 37 | 74.0% (61.8% - 86.2%) | 81 | 23.8% (19.2% - 28.3%) |
|  | Positive | 9 | 18.0% (7.4% - 28.6%) | 101 | 29.6% (24.8% - 34.5%) |
| Total |  | 50 |  | 341 |  |
|  |  |  |  |  |  |
| **ventilation** | Negative | 0 | 0.0% (0.0% - 0.0%) | 2 | 3.7% (-1.3% - 8.7%) |
|  | Neutral | 1 | 25.0% (-17.4% - 67.4%) | 5 | 9.3% (1.5% - 17.0%) |
|  | Positive | 3 | 75.0% (32.6% - 117.4%) | 47 | 87.0% (78.1% - 96.0%) |
| Total |  | 4 |  | 54 |  |
|  |  |  |  |  |  |
| **Pfizer** | Negative | 21 | 20.4% (12.6% - 28.2%) | 135 | 60.8% (54.4% - 67.2%) |
|  | Neutral | 76 | 73.8% (65.3% - 82.3%) | 68 | 30.6% (24.6% - 36.7%) |
|  | Positive | 6 | 5.8% (1.3% - 10.4%) | 19 | 8.6% (4.9% - 12.2%) |
| Total |  | 103 |  | 222 |  |
|  |  |  |  |  |  |
| **jab** | Negative | 16 | 22.2% (12.6% - 31.8%) | 141 | 65.9% (59.5% - 72.2%) |
|  | Neutral | 36 | 50.0% (38.5% - 61.5%) | 46 | 21.5% (16.0% - 27.0%) |
|  | Positive | 20 | 27.8% (17.4% - 38.1%) | 27 | 12.6% (8.2% - 17.1%) |
| Total |  | 72 |  | 214 |  |
|  |  |  |  |  |  |
| **mask** | Negative | 32 | 13.0% (8.8% - 17.1%) | 199 | 22.4% (19.7% - 25.2%) |
|  | Neutral | 144 | 58.3% (52.2% - 64.4%) | 106 | 12.0% (9.8% - 14.1%) |
|  | Positive | 71 | 28.7% (23.1% - 34.4%) | 582 | 65.6% (62.5% - 68.7%) |
| Total |  | 247 |  | 887 |  |
|  |  |  |  |  |  |
| **vaccine** | Negative | 108 | 19.2% (16.0% - 22.5%) | 378 | 44.0% (40.6% - 47.3%) |
|  | Neutral | 357 | 63.5% (59.5% - 67.5%) | 244 | 28.4% (25.4% - 31.4%) |
|  | Positive | 97 | 17.3% (14.1% - 20.4%) | 238 | 27.7% (24.7% - 30.7%) |
| Total |  | 562 |  | 860 |  |
|  |  |  |  |  |  |
| **Novavax** | Negative | 0 | 0.0% (0.0% - 0.0%) | 3 | 100.0% (100.0% - 100.0%) |
|  | Neutral | 15 | 88.2% (72.9% - 103.6%) | 0 | 0.0% (0.0% - 0.0%) |
|  | Positive | 2 | 11.8% (-3.6% - 27.1%) | 0 | 0.0% (0.0% - 0.0%) |
| Total |  | 17 |  | 3 |  |
|  |  |  |  |  |  |
| **booster** | Negative | 10 | 6.9% (2.8% - 11.0%) | 75 | 40.1% (33.1% - 47.1%) |
|  | Neutral | 103 | 71.0% (63.6% - 78.4%) | 30 | 16.0% (10.8% - 21.3%) |
|  | Positive | 32 | 22.1% (15.3% - 28.8%) | 82 | 43.9% (36.7% - 51.0%) |
| Total |  | 145 |  | 187 |  |
|  |  |  |  |  |  |
| **mandate** | Negative | 34 | 39.1% (28.8% - 49.3%) | 85 | 66.4% (58.2% - 74.6%) |
|  | Neutral | 45 | 51.7% (41.2% - 62.2%) | 16 | 12.5% (6.8% - 18.2%) |
|  | Positive | 8 | 9.2% (3.1% - 15.3%) | 27 | 21.1% (14.0% - 28.2%) |
| Total |  | 87 |  | 128 |  |
|  |  |  |  |  |  |
| **n95** | Negative | 2 | 25.0% (-5.0% - 55.0%) | 0 | 0.0% (0.0% - 0.0%) |
|  | Neutral | 6 | 75.0% (45.0% - 105.0%) | 2 | 5.6% (-1.9% - 13.0%) |
|  | Positive | 0 | 0.0% (0.0% - 0.0%) | 34 | 94.4% (87.0% - 101.9%) |
| Total |  | 8 |  | 36 |  |
|  |  |  |  |  |  |
| **respirator** | Negative | 0 | 0.0% (0.0% - 0.0%) | 0 | 0.0% (0.0% - 0.0%) |
|  | Neutral | 20 | 90.9% (78.9% - 102.9%) | 0 | 0.0% (0.0% - 0.0%) |
|  | Positive | 2 | 9.1% (-2.9% - 21.1%) | 1 | 100.0% (100.0% - 100.0%) |
| Total |  | 22 |  | 1 |  |
|  |  |  |  |  |  |
| **lockdown** | Negative | 36 | 35.0% (25.7% - 44.2%) | 23 | 54.8% (39.7% - 69.8%) |
|  | Neutral | 60 | 58.3% (48.7% - 67.8%) | 10 | 23.8% (10.9% - 36.7%) |
|  | Positive | 7 | 6.8% (1.9% - 11.7%) | 9 | 21.4% (9.0% - 33.8%) |
| Total |  | 103 |  | 42 |  |
|  |  |  |  |  |  |
| **iso/lation** | Negative | 9 | 18.0% (7.4% - 28.6%) | 2 | 10.0% (-3.1% - 23.1%) |
|  | Neutral | 34 | 68.0% (55.1% - 80.9%) | 1 | 5.0% (-4.6% - 14.6%) |
|  | Positive | 7 | 14.0% (4.4% - 23.6%) | 17 | 85.0% (69.4% - 100.6%) |
| Total |  | 50 |  | 20 |  |
|  |  |  |  |  |  |
| **immunity** | Negative | 6 | 13.0% (3.3% - 22.8%) | 35 | 35.4% (25.9% - 44.8%) |
|  | Neutral | 32 | 69.6% (56.3% - 82.9%) | 24 | 24.2% (15.8% - 32.7%) |
|  | Positive | 8 | 17.4% (6.4% - 28.3%) | 40 | 40.4% (30.7% - 50.1%) |
| Total |  | 46 |  | 99 |  |

**Table S5**

*Human-coded sentiment analysis by combined ‘vaccine’ and ‘mask’ search terms of entire dataset*

| **Search term** | **Sentiment** | **Frequency** | **Proportion (95% CI)** | **Frequency** | **Proportion (95% CI)** |
| --- | --- | --- | --- | --- | --- |
|  |  | **GA** |  | **X** |  |
| **Vaccine terms combined** | Negative | 158 | 18.1% (15.5% - 20.6%) | 827 | 49.5% (47.1% - 51.9%) |
|  | Neutral | 576 | 65.8% (62.7% - 69.0%) | 449 | 26.9% (24.8% - 29.0%) |
|  | Positive | 141 | 16.1% (13.7% - 18.6%) | 394 | 23.6% (21.6% - 25.6%) |
| Total |  | 875 |  | 1670 |  |
|  |  |  |  |  |  |
| **Vaccine terms including ‘booster’** | Negative | 168 | 16.5% (14.2% - 18.7%) | 902 | 48.6% (46.3% - 50.8%) |
|  | Neutral | 679 | 66.6% (63.7% - 69.5%) | 479 | 25.8% (23.8% - 27.8%) |
|  | Positive | 173 | 17.0% (14.7% - 19.3%) | 476 | 25.6% (23.6% - 27.6%) |
| Total |  | 1020 |  | 1857 |  |
|  |  |  |  |  |  |
| **Mask terms combined** | Negative | 34 | 12.3% (8.4% - 16.1%) | 199 | 21.5% (18.9% - 24.2%) |
|  | Neutral | 170 | 61.4% (55.6% - 67.1%) | 108 | 11.7% (9.6% - 13.8%) |
|  | Positive | 73 | 26.4% (21.2% - 31.5%) | 617 | 66.8% (63.7% - 69.8%) |
| Total |  | 277 |  | 924 |  |

**Table S6**

*Latent Dirichlet Allocation (LDA) topic modelling results for Google alerts dataset*

|  | **Topic summary GA dataset** | **Top Words** |
| --- | --- | --- |
| 1 | Public attitudes toward vaccines and masks | vaccine, people, vaccines, vaccination, public, mask |
| 2 | COVID-19 mitigation: mask usage and vaccination | masks, vaccine, covid, vaccination, vaccines, booster |
| 3 | COVID-19 protection and boosters | covid, masks, vaccine, mask, booster, jab |
| 4 | Australian COVID-19 response | covid, vaccine, health, booster, australian, vaccination |
| 5 | Intentions regarding public health measures | covid, vaccine, mask, lockdown, will, pfizer |

**Table S7**

*Latent Dirichlet Allocation (LDA) topic modelling results for X dataset*

|  | **Topic summary X dataset** | **Top Words** |
| --- | --- | --- |
| 1 | Discourse on mask wearing and COVID-19 vaccination | masks, covid, mask, jab, just, vax |
| 2 | Individual attitudes towards masks and vaccination | mask, people, vax, never, one, wear |
| 3 | COVID-19 choices including vaccine brands | vaccine, covid, pfizer, wearing, mask, vax |
| 4 | Intention to comply with PHM | mask, vaccine, covid, masks, just, will |
| 5 | Current perspectives on mask wearing and vaccination | vaccine, people, now, pfizer, wearing, mask |

**Figure S1**

GA dataset word cloud


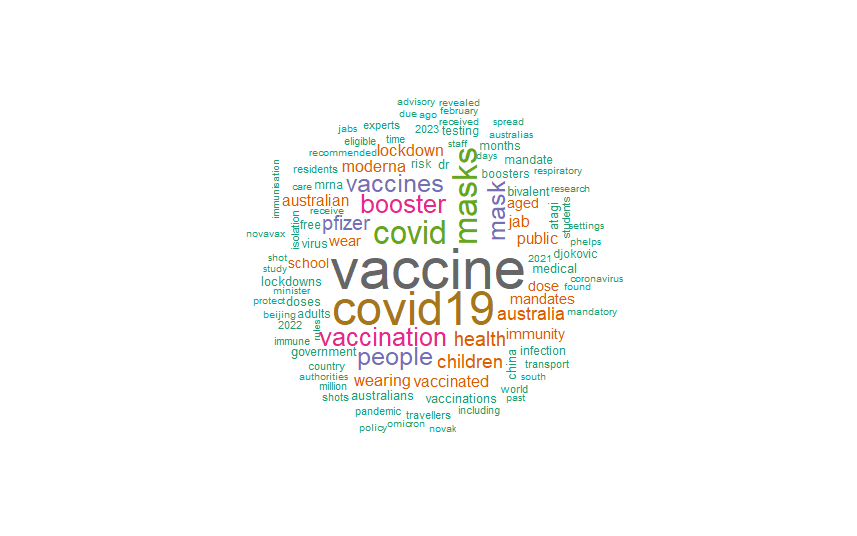


**Figure S2**

X dataset word cloud

**
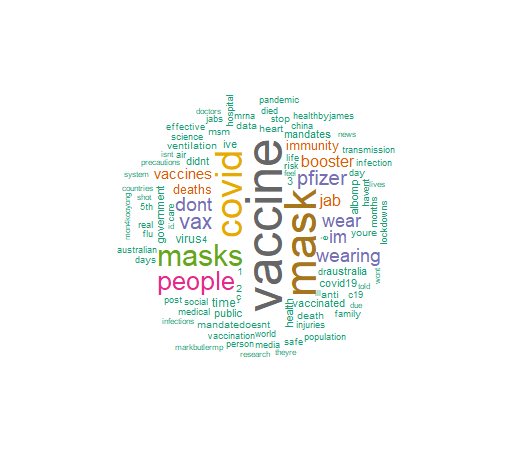
**

**Table S8**

*Results of the human-coded thematic analysis of positive and negative GA results and tweets*

a) Themes from Positive GA

| **Theme** | **Description** | **Example of a representative GA report** |
| --- | --- | --- |
| health professionals recommend masks |  | *Millions of Australians have been warned to wear face masks as the nation's latest COVID wave peaks over the busy Christmas period.* |
| masks recommended on transport to protect |  | *Australia scrapped its airline mask mandates in September, but global health officials are encouraging passengers to reconsider as the latest Omicron subvariant spreads rapidly.*  *It means masks are recommended in healthcare settings, on public transport, in crowded indoor settings.* |
| encourage vaccination in at-risk groups |  | *... the importance of stepping up vaccination coverage, including booster doses, particularly for vulnerable groups such as older persons.*  *Aside from the government and GPs, family members have an important role in encouraging senior citizens to get vaccines or booster shots against COVID-19.* |
| health professionals recommend vaccines and boosters |  | *Top health officials have also asked people to get vaccinated and take booster doses.* |
| positive impact of vaccines on economy | Reports on modelling and peer-reviewed papers | *The vaccine rollout saved the national economy $181bn in potential damage that could have been inflicted by ongoing lockdowns if a jab had been delayed.*  *COVID-19 vaccinations are estimated to have contributed to large positive effects for tourism exports ($28 billion) and education exports ($26 billion) exports, employment (142,000 jobs) and government finances ($259 billion)* |
| boosters are protective |  | *Professor discusses the importance of COVID booster shots, saying the virus is still a serious disease for some people.*  *Booster effectiveness peaked at approximately four weeks after receipt of booster and waned afterward. Hence, a booster dose is recommended for older patients to restore immunity.* |
| Anti-vax conspiracy | Addressing current anti-vax conspiracies | *Peters's pseudo-documentary, Died Suddenly, has been described as a “tsunami of anti-vax misinformation and conspiracy theories”.*  *A bizarre anti-vax conspiracy theory emerged in the US last week around NFL star Damar Hamlin.* |

b) Themes from negative GA

| **Theme** | **Description** | **Example of a representative GA report** |
| --- | --- | --- |
| Vaccines cause injuries and death |  | *Thousands of Australians suffering from COVID-19 vaccine injury feel they are “not being heard” or treated fairly by the government.*  *Former MP and vaccine advocate Dr Kerryn Phelps recently revealed that she and her partner both suffered significant neurological side effects.*  *Dr Kerryn Phelps has broken her silence about a “devastating” Covid vaccine injury, slamming regulators for “censoring” public discussion.*  *The mum of a “fit and healthy” 21-year-old from Melbourne who died after taking the Moderna vaccine has blasted authorities for mandating Covid-19 vaccines.* |
| Vaccine mandates have been harmful | Reporting on the negative impacts of vaccine mandates | *A mother has been denied the opportunity to receive a heart transplant because she is not vaccinated for Covid-19, despite having a medical exemption.*  *Coles is the only major supermarket in Australia that continues to employ discriminatory Covid vaccination mandates for workers.*  *Over 200 firefighters in New South Wales and Victoria are being forced to "stay away from saving lives" because of ongoing vaccine mandates.* |
| Masks have been harmful | Masks impacted babies development  Masks are not effective | “So people were very worried about face masks and the effect they would have on how infants are learning about human faces.” Oakes, an expert on ...  *People that had lessened lockdown and lessened mask measures did much better in all cause and mortality.* |
| Lockdowns have been harmful | Lockdowns had a negative impact on school children  Lockdowns caused suffering and economic harm | *The classroom mask mandates are no more, but experts are concerned over schoolchildren getting over behavioural and emotional habits developed during learning at home.*  *A top infectious disease expert warns it could be up to three years before Queenslanders fully regain their immunity.*  *Lockdowns were a euphemism for a wholesale shutting down of social and economic activities and putting entire populations under house arrest.* |

c) Themes from positive tweets

| **Theme** | **Description** | **Example of a representative tweet** |
| --- | --- | --- |
| Masks are effective | May quote scientific studies or anecdotes from personal experience | *Most studies support the efficacy of n95s for aerosolised material and surgical masks for droplet transfer*  *Wearing a mask saved my life during cancer treatment* |
| Masks protect the wearer | Reasoning including vulnerability to severe disease, due to others not wearing masks and exhibiting symptoms, framing it as the “smart/sensible” behaviour, as opposed to the “stupid/foolish” behaviour*. | *I don’t want to get sick, so I wear a mask, nothing wrong with that.*  *My daughter works with Covid patients and we wear a mask when she visits. My partner has cancer.*  *Lots of people coughing on the train and I’m the only one smart enough to wear a mask.* |
| Masks protect others | Wearing masks to protect others in the community who may be vulnerable to severe disease, including the elderly * or immunocompromised. Framed as “unselfish/considerate” behaviour compared to “selfish/irresponsible” behaviour | *Wearing a mask to protect the elderly and vulnerable is an act of kindness to others*  *It is selfish not to wear a mask to protect the health of our most vulnerable.* |
| Important to wear masks in specific contexts | Healthcare settings (including hospitals, GP practices and pharmacies)  Public transport (Including trams, buses, trains and planes)  Indoor or crowded settings | *People should wear masks in healthcare settings. GP clinics/pharmacies are full of sick people with no masks now.*  *I’m in hospital right now, no one is wearing masks. I can't believe this is happening.*  *I’m the only person wearing a mask on a packed train and people are coughing everywhere.*  *We can’t live it masks forever, but it makes sense to mask up in crowded shopping centres.* |
| Support for mask mandates | In specific settings such as healthcare, flights and public transport. | *Mandating masks on planes should be the bare minimum.*  *How about mask mandates in all healthcare setting, and on PT, taxis, and planes?* |
| Benefits of vaccines outweigh the risks |  | *Severe adverse events are rare but do happen. The COVID vax has prevented millions of deaths worldwide.*  *I’m sorry for people injured by the vaccine but the risk is insignificant compared to complications from Covid.* |
| Vaccines are effective | Save lives  Decrease severity | *My mother had a heart issue and caught covid. Without being vaccinated she would have died.*  *Without vaccines there would have been more infections and more mutations.*  *I’m so grateful… the vaccine has saved millions of lives globally.* |
| Want access to boosters | Keen to get 4^th^ or 5^th^ COVID-19 vaccine  Prefer updated bivalent | *I saw on the news that we might get 5^th^ jab in February – can’t come soon enough!*  *It would be nice to get an up-to-date bivalent – US and Canada are using BA4/5 and we are stuck with BA1.* |
| COVID-19 conspiracies are false | Mocking that all deaths are due to the vax now | *In 2019 when a bloke smoked and drank and died of a heart attack it was because of lifestyle, now it’s “because of the jab”.*  *In 50 years from now when someone dies … first tweet will be… did they have the jab?!!*  *Anecdotally I know two people who had vaccine reactions out of the hundreds of people I know who haven't – there is no conspiracy.* |
| Public health measures are important to protect the community | More important than economic interests  Encourage others to continue with preventative practices (mask wearing, isolation, hand hygiene, social distancing, vaccination) even though all restrictions have eased.  Government should provide free access to NPIs such as masks, RAT tests | *The government only want to keep businesses happy, undermining mitigation efforts, no responsibility for public safety!*  *We have to protect ourselves, covid deaths are up, mask on public transport, vaccines, antivirals, social distancing.*  *We need to government to spend more money on free PCRs and RATs, masks & vaccines to end this pandemic.* |

d) Themes from negative tweets

| **Theme** | **Description** | **Example of a representative tweet** |
| --- | --- | --- |
| COVID-19 vaccines cause injuries and deaths | Vaccine injured  Side effects (including hormonal)  Sudden death  Media hiding numbers | *Vaccine injury and deaths outnumber actual deaths FROM Covid*  *My neighbour told me that her son’s friend went down to the local shopping centre to get vax, dies 15 minutes later.*  *I saw a doctor speaking about all the hormonal side effects from the vaccine – huge menstrual changes and menopause.*  *You'll never hear even so much as a peep from the mainstream media about why so many people have #diedsuddenly.* |
| COVID-19 vaccine is experimental |  | *Why is the government pushing a vax that hasn’t been approved and is experimental?*  *This is an untested vaccine that has caused more harms than the virus.* |
| Brainwashed people follow government advice | Covid vax = cult, junkies  Smart people did not get vaccinated | *The vaccines cultists are talking about how many lives were saved – if you still believe that, you should get boosted right now.*  *Scientists came up with this vaccine in 10 weeks, and people still believe it’s safe, talk about being brainwashed.*  *The sheep are rolling up their sleeves for their 5^th^ jab.*  *I don’t inject poison into my body, unlike the vax junkies.*  *The smartest people in the country were those who refused to get the jab.* |
| Vaccine is ineffective |  | *The jab doesn’t work anyway… Pfizer admitted it.*  *Vaccines don’t confer immunity – it was useless from day 1.* |
| Conspiracy theories | Destroys immunity  5G  Causes HIV/AIDS  Increases infections | *The vaccine clinical data shows that it destroys the body’s natural immune system.*  *Authorities in the UK were told that the vaccine causes an increased risk of COVID and each booster makes the body less able to cope with infection.*  *There will be mass deaths of the vaccinated by weaponised 5G.*  *The Government wants us to get a COVID-19 Booster every 3 months because the vaccine is causing a new form of AIDS.*  *Infection rates are surging due to the vaccination mandate* |
| Masks are ineffective |  | *Masks do nothing at a population level.*  *Facemasks do not stop the transmission of covid-19.*  *I have never worn a mask and haven’t caught covid.* |
| Reasons people wear masks are | Virtue signalling  Stupid people wear masks  Social pressure | *Why are people so obsessed with wearing masks? It’s just virtue signalling?*  *Dudes still wearing masks here, who knows why. Only morons think they work.*  *I feel sad for people who still pretend that the magic mask protects them.* |
| Mandates are ineffective | Mandates are not effective  Attack on individual freedom/loss of freedom  Discrimination | *Not one country has made mask mandates work. Look at Singapore, South Korea and Hong Kong – strict mask mandates and more cases per capita than the US and UK.*  *Mask mandates cause infections and mental illness.*  *The medical system is discriminating against unvaccinated people – woman was refused a heart transplant – due to coercive vaccine mandate. It’s cruel!*  *It’s 2023 and vaccine mandates still exist – I used to feel free in this country.* |
| Having repeated boosters is for the brainwashed | Fool/brainwashed to get booster  Sheep | *Take your 12^th^ booster and stay indoors little buddy.*  *My parents are double boosted mask-wearing sheep. Can’t accept the truth when it’s in front of them.* |
| Boosters are ineffective |  | *Studies show that the boosters don’t work, but the vaccine companies are still pushing them.*  *Boosters are useless – nothing beats natural immunity.* |
| Pharma companies are only interested in profits |  | *Covid vaccines don’t work against the new variant. Not that Pfizer cares – they’re only interested in their profits.* |
| Pharma conspiracy | Virus was created for the vaccine | *The virus was created so their pre made killer vaccines could be jabbed into millions of arms – so unethical!* |
| Lockdowns caused harm | Economic  Trauma  Ineffective | *Lockdowns achieved nothing but pain and suffering. Many in Victoria are still suffering from PTSD.*  *Lockdowns did more harm than good with immunity debt as a result.*  *Lockdowns destroyed the economy and cost people their jobs.* |
