# Supplementary material for: Comparison of Artificial Intelligence Tools With Human Coding for Sentiment, Topic, and Thematic Analysis Tasks of Public Health Datasets During the COVID-19 Pandemic in Australia: Case Study
Source: Online J Public Health Inform. 2026 Apr 7;18:e80824. doi: 10.2196/80824 (PMC13063369; doi:10.2196/80824)
Supplement: Multimedia Appendix 2 [file ojphi-v18-e80824-s002.docx]

install.packages(c("tm", "topicmodels", "readr"))

library(readr)

library(tm)

library(topicmodels)

data <- read_csv("GA topic.csv")

texts <- data$text

corpus <- VCorpus(VectorSource(texts))

corpus <- tm_map(corpus, content_transformer(tolower))

corpus <- tm_map(corpus, removePunctuation)

corpus <- tm_map(corpus, removeNumbers)

corpus <- tm_map(corpus, removeWords, stopwords("english"))

dtm <- DocumentTermMatrix(corpus)

rowTotals <- apply(dtm, 1, sum)

dtm <- dtm[rowTotals > 0, ]

k <- 5

lda_model <- LDA(dtm, k = k, control = list(seed = 1234))

print(terms(lda_model, 6))
